# Risk of exotic disease introduction and propagation in the Austrian swine trade network

**DOI:** 10.1016/j.isci.2026.114868

**Published:** 2026-02-02

**Authors:** Gavrila Amadea Puspitarani, Hannah Schuster, Ewan Colman, Amélie Desvars-Larrive

**Affiliations:** 1 Clinical Department for Farm Animals and Food System Science, University of Veterinary Medicine Vienna, Vienna, Austria; 2 Complexity Science Hub, Vienna, Austria; 3 University of Economics and Business, Vienna, Austria; 4 Bristol Medical School, University of Bristol, Bristol, UK

**Keywords:** animal health management, animal science, food animal veterinary medicine, porcine epidemiology

## Abstract

Importation of live pigs poses a significant risk for introducing exotic diseases, threatening animal health, welfare, and food security. Using daily Austrian pig movement records from 2021, we modeled the introduction of an infectious disease. Within-holding infection dynamics were simulated with a stochastic susceptible-exposed-infectious-removed (SEIR) with ASF-like parameters; between-holding transmission occurred via direct trade and indirect local spread within 5-km radius. Across simulations, the epidemic affected 0.2% of pigs and 2% of holdings, reaching 10% of municipalities. Most holding-to-holding transmission was short-distance (54.9% intra-municipal; inter-municipal transmission averaged 7.8 km), but rare long-distance events (mean 5.6 events per simulation; >2 SD above mean trade distance) facilitated large-scale outbreaks. Early-stage projections predicted final size and progression more precisely than later forecasts, supporting timely targeted interventions. Static networks overestimated affected municipalities by 8.9-fold. The first 40 days were critical for epidemic control when introduction occurred in a low-trade period (January), shrinking to 20 days during high-trade periods (April).

## Highlights

• Highest-import municipality (M1514) is a likely entry point, import ∼30% more
• Long-distance transmission seed distant cluster, driving ∼20% of outbreak spread
• Static networks ignoring timing overestimate affected municipalities by 8.9-fold
• Control windows: 20/40 days; long-distance transmission probability rises after 100 days

## Graphical abstract

**Figure:**
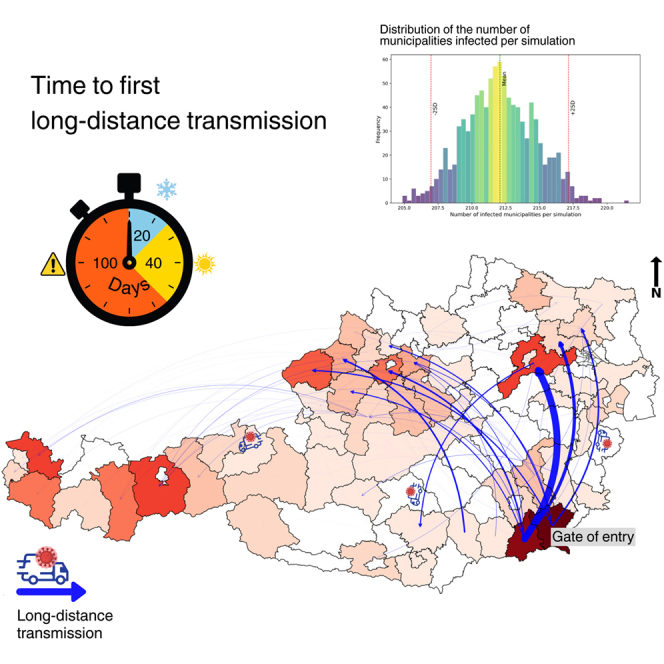

## Introduction

In Europe, millions of live animals are transported annually for various purposes, increasing the potential for communicable diseases to spread across the European Union (EU). Interstate movements are particularly at risk for the introduction of exotic pathogens into disease-free regions.^1^^,^^2^^,^^3^^,^^4^ A notable example is the introduction of foot-and-mouth disease (FMD) into the Netherlands from Irish calves, which further spread into mainland Europe.^5^ Despite such risks, few surveillance systems use international livestock movement data to monitor and mitigate the risks of disease introduction,^6^^,^^7^ highlighting a critical gap in the current biosecurity framework.

Pigs are among the most frequently traded livestock in Austria, with over 1.9 million movements recorded between 2015–2021,^8^ heightening the risk of introducing infectious diseases and facilitating their subsequent spread.^1^ In Austria, significant concerns include African swine fever (ASF) and classical swine fever (CSF), both highly contagious viral diseases that can cause severe economic losses in the pig industry.^9^^,^^10^^,^^11^ Despite Austria’s current ASF-free status, the disease is present in several neighboring countries,^12^ elevating the risk of transboundary spread via legal or illegal movement of infected animals or contaminated products. Understanding the vulnerabilities of pig production systems in relation to trade dynamics, notably importations, can provide critical insights for designing targeted animal health surveillance systems.

At farm level, the incidence of infectious diseases is positively associated with the number of incoming contacts, i.e., the number of farms from which animals are purchased.^13^ Regions located downstream of major animal mobility flows are often at increased risk of disease introduction, while holdings with numerous outgoing movements can act as key propagators of infection.^14^^,^^15^^,^^16^ Disease propagation typically involves a combination of short-distance spread and long-distance dispersal events, closely tied to the structure of livestock mobility networks.^4^^,^^17^ Short-distance spread affects nearby holdings through indirect paths (e.g., arthropod vectors^18^^,^^19^ and fomites^20^^,^^21^^,^^22^^,^^23^^,^^24^^,^^25^^,^^26^) or via direct contact, such as the introduction of infected animals via transfer.^17^^,^^27^

In the EU, animal movements must be reported,^28^ allowing for traceability, while regulating livestock movements during outbreaks provides an effective means of controlling disease spread.^29^^,^^30^ Livestock mobility can be modeled using origin-destination (OD) matrices, with or without time attributes.^31^^,^^32^ These data can be represented as a directed network where communities, such as holdings, spatial zones (e.g., grid cells^33^^,^^34^), or administrative regions,^16^ form nodes and edges represent trading operations (“flow”) between them. Additionally, edges can be weighted based on the intensity of animal movements between communities, e.g., using trade volume or frequency, providing a more accurate representation of movement dynamics.^8^^,^^35^

To better understand and predict infectious disease dynamics, compartmental models are widely used in epidemiology. These models divide the population into distinct compartments according to disease status, such as susceptible (S), exposed (E), infected (I), and recovered/removed (R), forming the SEIR model.^36^^,^^37^ The integration of pig mobility data with compartmental models has been instrumental in simulating the spread of infectious diseases such as FMD,^38^^,^^39^ porcine reproductive and respiratory syndrome (PRRS),^40^^,^^41^^,^^42^ CSF,^43^^,^^44^^,^^45^ ASF,^46^^,^^47^ and porcine epidemic diarrhea (PED).^48^

Beyond understanding disease spread, a key application of network models is to inform and optimize outbreak control. Sophisticated mathematical frameworks, such as optimal control theory,^49^ are increasingly applied to epidemiological networks to derive cost-effective intervention strategies over time, for instance, by optimizing vaccination allocation^50^^,^^51^ and quarantine strategies.^52^ While these approaches are powerful for determining “how much” and “how to” intervene, they first need identifying “who” to target and “when” to act. Our study focuses on these two foundational steps.

While the epidemiological importance of trade networks is well-established in other contexts, their specific dynamics of the Austrian pig movement network remain unexplored. The objective of this study was to explore likely scenarios that would follow the introduction of a disease into the pig population of Austria. Our model is the first to integrate high-resolution pig movements data from Austria into an epidemiological model, yielding several context-specific policy-relevant findings. The novel inclusion of temporally resolved data show that static networks substantially overestimate outbreak size and critical intervention windows are seasonally dependent. We characterize long-distance infection jumps, identify specific municipalities as transmission hubs, and model disease spread from a data-driven high-risk entry point. These findings provide critical insights to guide targeted disease prevention strategies and optimize resource allocation for outbreak management in Austria.^1^^,^^53^

## Results

### Mobility flow patterns in the static pig trade network

#### Strong correlation between inflow and outflow in the domestic pig mobility network

**Figure 1:**
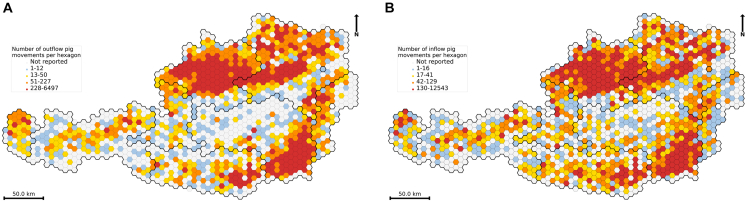
Flow of pig trades aggregated to hexagonal grid cells in Austria in 2021 (A) Outflow trade frequencies. (B) Inflow trade frequencies. Each hexagon represents a 5 km^2^ area. Municipal-level trade frequencies were aggregated to hexagons by summing movements from municipalities whose centroids fall within each hex. Hexagons are color-coded based on quantiles of the flow distribution, highlighting variations of trade activity across the country. Hexagons with no flow are shown in white. Thick black lines represent federal state boundaries. Data are derived from a total movement record of 250,137.

Analyses revealed that annual outflow per municipality ranged from 1 to 3,458, while inflow ranged from 1 to 10,267 trades (Figure 1). High-flow areas were concentrated in the federal states of Upper Austria and Styria. The Spearman correlation test revealed a strong positive relationship (*r*_*s*_ = 0.76, *p* value = 1 < 10^−300^) between inflow *F*_*in*_ and outflow *F*_*out*_. This highlights the existence of “hubs” characterized by both a high vulnerability to disease introduction (sinks) and a large potential for widespread transmission (amplifiers).

#### Importation patterns and high-risk entry point

In 2021, Austria recorded 4,160 pig importation events, primarily sourced from Germany (89%), followed by Hungary (3.4%), Slovenia (3.3%), Croatia (2%), the Netherlands (1.8%), the Czech Republic (0.5%), and Denmark (0.1%). These imports were distributed across 64 municipalities, with per-municipality event counts ranging from 1 to 1,243. The municipality M1514 emerged as the highest risk entry point, receiving 1,243 importation events (29.7% more than the second-highest municipality; 958 events) that brought 86,461 pigs into the region. This volume represented 18.6% of all pigs imported into Austria, with an average of 69.6 pigs per import event. With 25 active holdings, M1514 was identified as a critical hotspot for potential exotic disease introduction via live pig movements.

**Figure 2:**
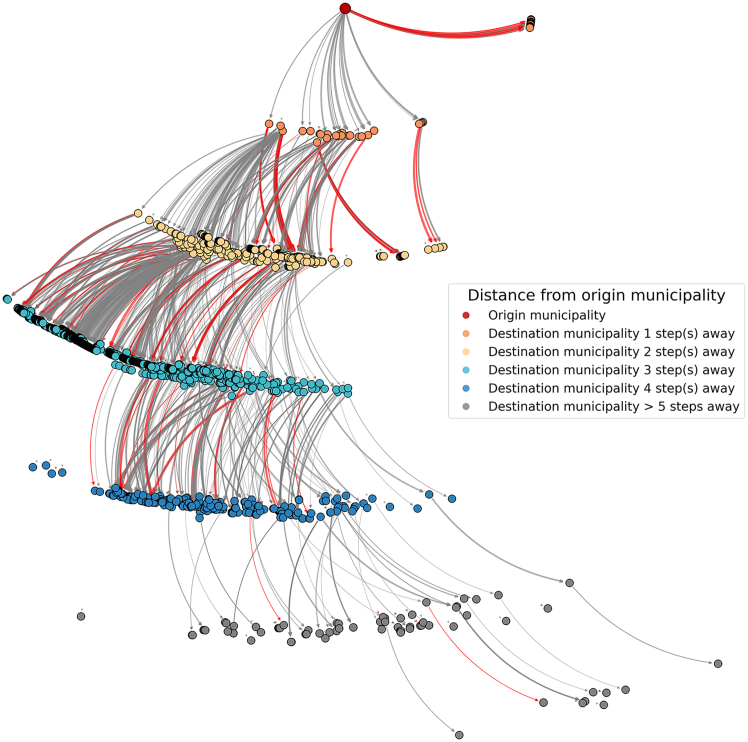
Potential propagation network from M1514 (highest-import municipality), Austria, 2021 The network representation illustrates pig trade pathways originating from M1514, Austria. Nodes represent municipalities and are color-coded based on their stepwise distance from the origin (M1514, which is shown in dark red), while municipalities 1 to more than 5 steps (i.e., trades) away are shaded as indicated in the legend. Directed edges represent trade movements, with red edges indicating slaughter-related connections and gray edges representing other trade types.

Using 2021 Austrian pig movement data, we constructed a propagation network to model potential disease spread originating from M1514 (Figure 2). This network encompassed all municipalities that could have received pigs directly or indirectly from this high-risk entry point. We found that: (1) M1514 was linked to 44 municipalities via direct movements (*k* = 1); and (2) through pathways of five or more trade steps (*k* ≥ 5), 1,839 municipalities (89% of Austria’s total) were reachable (Figure S2).

**Figure 3:**
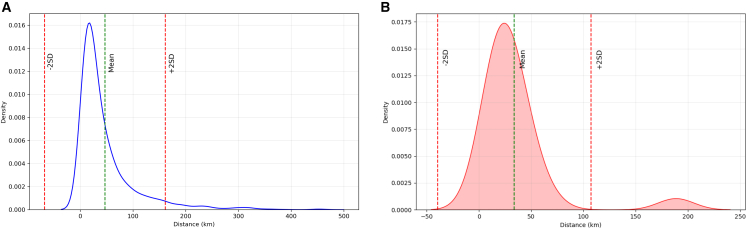
Euclidean distance distribution of domestic pig movements in the potential propagation network from M1514 (highest-import municipality), Austria, 2021 Distances are in kilometers (km) and correspond to trade distance between municipalities within the propagation network. (A) Distribution of Euclidean distances for all trades originating from M1514. The green dashed line represents the mean trade distance (46.8 ± 57.3 (SD) km). The dashed red line on the right side indicates the threshold for long-distance movements (161.4 km, i.e., mean +2SD). (B) Distance distribution for pig trades at step one (*k* = 1). The green dashed line represents the mean trade distance at *k*1 (33.8 ± 36.7 (SD) km).

The mean Euclidean trade distance between municipalities in the propagation network was 46.8 ± 57.3 (SD) km (Figure 3A). We defined long-distance trades as those exceeding 161.4 km (mean +2SD). The majority (39, 88.6%) of these M1514-directly connected municipalities were located within Styria, while two were in Upper Austria, approximately 200 km away, two were in Burgenland, and one in Carinthia.

The Euclidean trade distances of M1514-directly connected municipalities in the propagation network exhibited a bimodal distribution (Figure 3B). Most of the directly connected municipalities were located at distances centered around a mean of 33.8 ± 36.7 km from M1514.

A similar pattern was observed for propagation steps *k* ∈ [2, 5] (Figure S3). The mean inter-municipality Euclidean distance for these steps ranged from 27.7 km to 55.5 km, indicating that most trades—and thus potential transmission pathways—occurred over relatively short distances, i.e., primarily between neighboring municipalities. The monthly frequency of long-distance trade events (Figure S5) increased from January, peaked in May, and subsequently declined to the lowest levels in late autumn and winter.

Finally, we also calculated that by the third step (*k* = 3) in the propagation network, 1,283 municipalities (60.7% of Austrian municipalities) were reached.

### Epidemic size and dynamics

We modeled disease spread in Austria’s pig production system, simulating within-farm transmission, local indirect spread, and movement-mediated transmission. The model was parameterized to mimic ASF dynamics. Two scenarios were tested: one initiating in January and another in April, each starting at a randomly selected holding in the high-import municipality (M1514).

**Figure 4:**
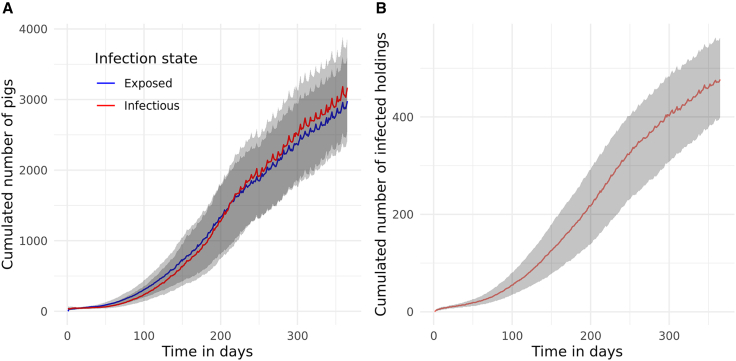
Temporal dynamics of epidemic size over a 365-day simulation following the hypothetical introduction of an ASF-like disease in Austria on 1st January 2021 One holding, located in municipality M1514 (the highest-import municipality), was randomly selected as the origin of the outbreak. (A) Median number of pigs in the exposed *E* (blue line) and infectious *I* (red line) compartments across all municipalities. (B) Median number of infected holding (red line) across municipalities. A holding was considered infected if it contained at least one pig in either the exposed *E* or infectious *I* compartment. Once a holding became infected, it remained infected for the duration of the simulation. Shaded gray areas indicate the 95% confidence intervals (CI), reflecting variability across 1,000 simulations. The widening of the CIs over time indicates increasing uncertainty in the model's predictions as the epidemic progresses.

In all simulations, the number of exposed (*E*) and infectious (*I*) pigs increased steadily over time. The infectious pigs population peaked at 3,183 on day 359 while exposed pigs reached their maximum of 2,978 on day 365 (Figure 4A).

**Figure 5:**
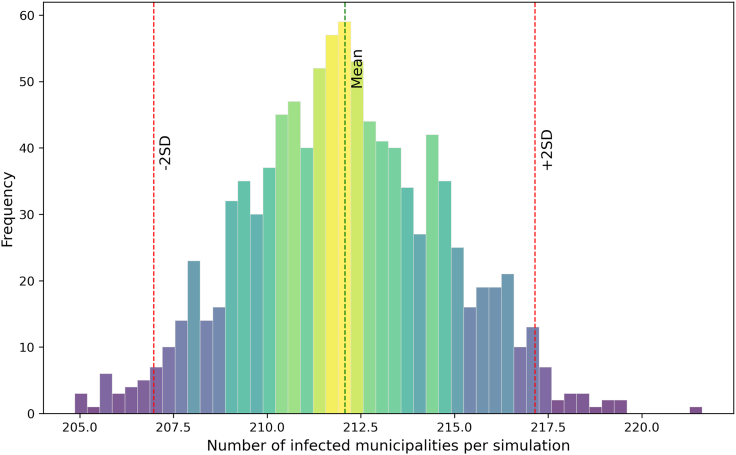
Distribution of the number of municipalities infected per simulation, after hypothetical introduction of an ASF-like disease in Austria on 1 January 2021 One holding, located in the municipality M1514 (the highest-import municipality), was randomly selected as the origin of the outbreak. The dashed green line represents the mean number of infected municipalities across 1,000 simulations (mean = 212). The red dashed lines represent the ±2 standard deviation (SD) from the mean [207.2–217.2].

**Figure 6:**
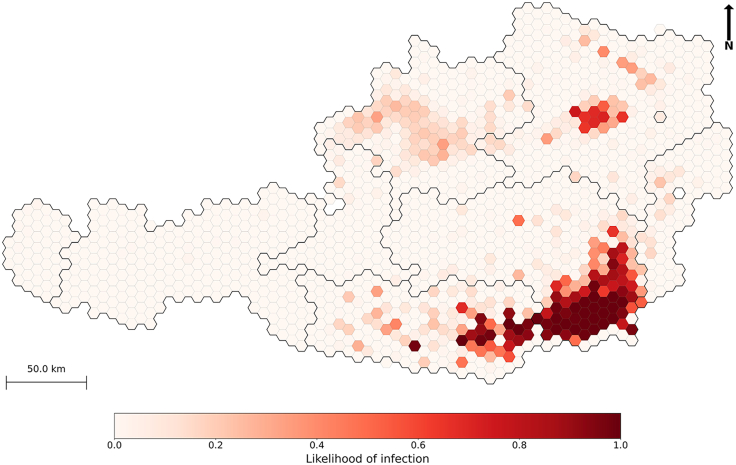
Hexagonal aggregation of infection likelihood across Austria following the hypothetical introduction of an ASF-like disease on 1 January 2021 One holding, located in the municipality M1514 (the highest-import municipality), was randomly selected as the origin of the outbreak. For each simulation iteration, we recorded whether a municipality was reached by the infection and then counted these occurrences. The resulting frequencies were normalized by the total number of iterations (1,000) to obtain likelihood values ranging from 0 (never reached) to 1 (always reached). These likelihood values were then averaged within each hexagonal cell (5 km radius). Data are presented as mean likelihood per cell. Thick black lines represent federal state boundaries.

The number of infected holdings exhibited an initial smooth increase in the first 25 days which transitioned to an exponential-like growth pattern, indicating a rapid rise in infections before stabilizing (Figure 4B). By the end of the simulation period, the number of infected holdings reached 477 (95% CI: [400–563]), representing approximately 2% (95% CI: [1.7–2.4]) of all trading holdings in Austria in 2021. An average of 212 (95% CI: [207.2–217.2]) municipalities (10%) were affected (Figure 5). The resulting likelihood of infection showed that the highest likelihoods were concentrated around the origin of initial outbreak (Figure 6). This estimate is substantially lower than the potential geographic range predicted by the analysis of the movement network (89% of all municipalities, Figure S2).

#### Epidemic size does not vary with incursion time

Given that trade flows exhibit marked seasonal variation—with volumes typically peaking in April^54^—we hypothesized that the timing of pathogen incursion may influence the subsequent epidemic size.

Within 30 days following the disease introduction, simulations initiated in January resulted in 62 exposed *E* and 50 infectious *I* pigs, whereas those initiated in April showed slightly lower counts, with 58 pigs in *E* and 47 pigs in *I* (Data S1). A similar trend was observed in the number of infected holdings, with 12 holdings infected in the January and April scenarios (Data S1).

Data S1. Comparison of epidemic dynamics at different seeding times in a PDF

Statistical analysis using Mood’s median test showed no significant difference between the two scenarios for the number of pigs in *E* (*p* value = 0.58) and *I* compartments (*p* value = 0.26) at *t* = 5 onward. Similarly, no significant difference was observed in the number of infected holding at *t* = 3 onward (all *p* value >0.1).

Results from the analysis of epidemic impact after the April disease incursion are presented in Data S1.

#### Infection jumps via long-distance trade

Transmission events predominantly occurred over short distances, with 54.9% (95% CI: 54.8%–55%) taking place within the same municipality. When transmission occurred between municipalities, the average inter-municipal distance was 7.8 ± 10.4 km, consistent with localized spatial spread. Of particular epidemiological significance were long-distance transmission events (>mean + 2SD, Figure 3B) as they have the potential to seed new, geographically distant clusters far from the outbreak origin, and substantially alter the spatial trajectory of the epidemic.^4^^,^^27^^,^^55^

We quantified the duration of the initial local spread phase (i.e., the period before the first long-distance jump). This phase persisted for an average of 143 days (95% CI: 139–147.2). For simulations initiated in January, the mean number of long-distance infection jumps per run was 5.6 ± 3.0, with a median of five (range: 0 to 22) (Figure S4). Our analysis of the relative contribution of transmission routes revealed that while only a small fraction of municipalities were directly seeded by a long-distance transmission event, the secondary clusters originating from these events accounted for 20.1% (95% CI: 19.0–21.1%) of all affected regions, with the remaining 79.9% (95% CI: 78.9–81.0%) attributable to local spread.

Municipalities serving as sources for long-distance transmission events exhibited heterogeneous outflow centrality values (total outgoing of pig movements), a metric representing the likelihood to spread infection via long-distance trade. Notably, municipalities M1491 (*λ* = 2.3), M1510 (0.4), and M0292 (0.4) showed the highest outflow, indicating they were the most probable source of such spreading events.

**Figure 7:**
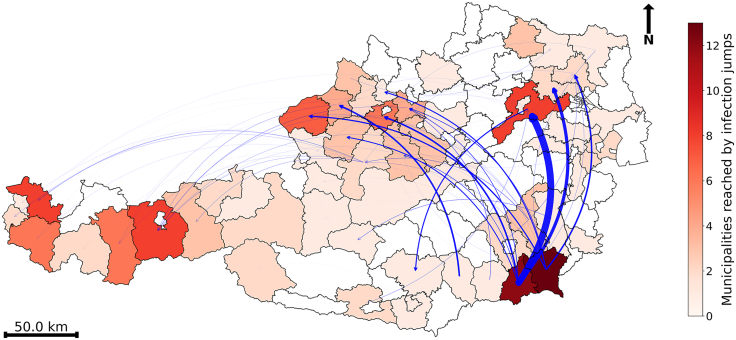
Districts involved in long-distance infection jumps and the likelihood of long-distance infection jumps following the hypothetical introduction of an ASF-like disease in Austria on January 1, 2021 One holding, located in the municipality M1514 (the highest-import municipality), was randomly selected as the origin of the outbreak. Analysis is aggregated to the district level to highlight broader transmission patterns while protecting personal data. Districts shown in red contain at least one municipality that participated in at least one long-distance infection jump, either as a source or destination, with color intensity reflecting the number of participating municipalities within each district (across 1,000 simulations). Districts shown in white did not participate in any long-distance infection jumps across all simulations. Directed blue arcs indicate long-distance infection jumps, with arrows pointing to the destination districts. Arc thickness is proportional to the likelihood of occurrence across simulations, highlighting critical connections within the network that may drive large-scale outbreaks.

Similarly, municipalities receiving long-distance transmission events exhibited heterogeneous inflow centrality values (total ingoing of pig movements). The most vulnerable municipalities included M0731 (*λ* = 0.7), M0797 (0.7), and M0720 (0.4). These municipalities were primarily concentrated in southeastern Austria, specifically in Styria and Carinthia, with additional hotspots in Lower Austria and Upper Austria (Figure 7).

The farthest recorded infection jump in our simulations occurred from a municipality in Lower Austria to a municipality in Vorarlberg, covering a distance of 501.3 km. However, the likelihood of this event was extremely low (0.001). The propagation of the disease to the distant region of Vorarlberg consistently occurred through multi-step transmission rather than a direct jump from the outbreak origin. All long-distance infections reaching Vorarlberg originated not from the initial hotspot (M1514) in Styria, but from subsequent foci in Upper Austria.

**Figure 8:**
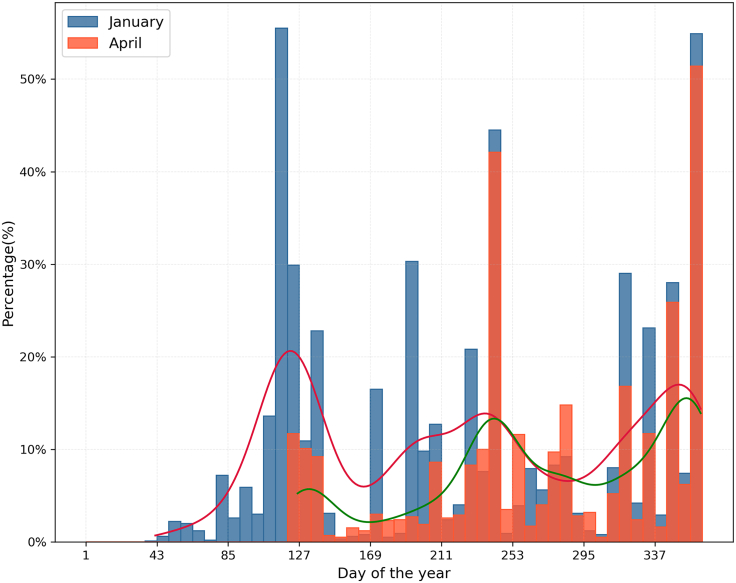
Temporal distribution of successful infections via long-distance trades following the hypothetical introduction of an ASF-like disease in Austria under January and April seeding scenarios The histogram shows the percentage of simulations (out of 1,000) in which a long-distance infection jump occurred within each time bin, with blue bars representing January seeding and orange bars representing April seeding. The outbreak originated from a randomly selected holding in the municipality M1514 (the highest-import municipality). The overlaid kernel density estimates (KDEs; red and green lines for January and April, respectively) highlight the temporal density of events and reveal distinct peaks at specific time intervals.

A critical window for local disease containment is the period between initial incursion and the first long-distance transmission event.^56^^,^^57^ To measure this, we reported the shortest time from incursion to the first long-distance infection jump across 1,000 simulations. When seeded in January, the earliest first long-distance infection jump occurred at day 42 after the incursion. In the following week (day 42–49), the probability of a long-distance infection jump to occur was 0.7%. In contrast, in the April seeding scenario, the earliest infection jump occurred at day 20 after disease introduction (calendar days 126–133). In the following week (day 20–27) the probability of occurrence of a long-distance infection jump was 11.7% (Figure 8). These results demonstrate that April-initiated outbreaks progressed to the long-distance transmission phase 2.1 times faster than January-initiated outbreaks.

Temporal analysis of the January seeding scenario revealed a multimodal pattern in long-range transmission risk, characterized by three distinct peaks in the probability of long-distance transmission events (Figure 8). The first and most prominent peak occurred between *t* = 113 − 120 days post introduction, at a probability of 55.5%. Two subsequent peaks were observed at *t* = 239 − 246 days (44.5%) and *t* = 358 − 365 days (54.9%), with the final peak coinciding with the simulation endpoint (day 365). Poisson regression indicated a statistically significant negative association between weekly overall trades and weekly infection jump events (0.01% decreases in expected infections per trade unit, *p* = 0.008) but also revealed extreme overdispersion (Deviance/df ≈ 152; Pearson *χ*^2^/df ≈ 190) (Data S1).

**Figure 9:**
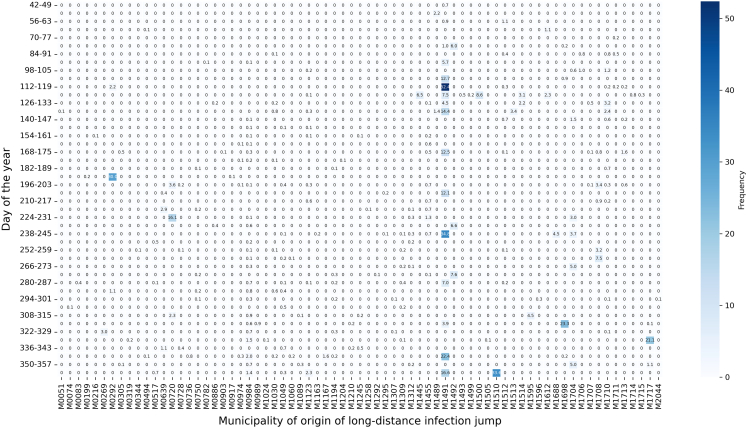
Temporal distribution of successful long-distance infection jumps in the propagation network following the hypothetical introduction of an ASF-like disease in Austria on January 1, 2021 The *x* axis displays the municipality codes. The intensity of the blue shading corresponds to the frequency of long-distance infection jumps, with darker shades indicating higher frequencies. Numbers within the cells denote the probability of successful infection jumps across 1,000 simulations for each municipality-time combination.

Municipalities exhibited varying contributions to long-distance infection jumps throughout the epidemic (Figure 9). For instance, at *t* = 113 − 120 days, municipality M1491 emerged as the most frequent source of long-distance infection jumps, contributing to 524 jumps. This municipality consistently served as a key source of long-distance transmission across multiple time frames. Similarly, at *t* = 190 − 197 days, municipality M0292 was associated with the highest number of long-distance infection jumps (300). The median timing for the infection to reach these “long-range spreaders” was 74 ± 37.7 days for M1491, 62.5 ± 50.1 days for M1510, and 132days ±51.9 for M0292.

Results from the temporal analysis of the April seeding scenario are presented in Data S1.

## Discussion

By simulating an ASF-like outbreak seeded at a single high-risk importation point (M1514), our study demonstrates how the timing of disease introduction and the structure of the domestic pig trade network jointly determine the speed and spatial extent of epidemic spread. Despite exploring only one entry scenario, the results reveal important dynamics: early-stage spread remains local for a limited period, after which the likelihood of long-distance dissemination increases sharply—especially when the introduction occurs during peak trade activity. Although rare, long-distance transmission events had a disproportionate impact on outbreak geography by establishing secondary clusters that subsequently spread the disease further. This is exemplified by the infection of Vorarlberg, which was not seeded directly from the outbreak origin but was relayed via the central hub of Upper Austria, highlighting the disproportionate role of amplifier regions in long-range dissemination. Municipalities in Upper Austria and Styria, characterized by high frequencies of both inflow and outflow of pigs, emerge as central hubs in this network. Their dual role as major receivers, making them vulnerable to disease incursion, and as distributors, positioning them as key transmission points, underscores their pivotal influence in shaping epidemic dynamics. These findings emphasize the importance of early detection and rapid response, particularly in highly connected municipalities that function as both receivers and major distributors within the trade network.

Studies have consistently shown a positive relationship between import activities and the risk of disease introduction.^3^^,^^27^ We identified M1514 as a critical high-risk importation point, which received the highest number of import events and a substantial proportion of the national import volume, serving as a potential epicenter where introduced infections could cascade through the network.

Integrating 2021 pig mobility networks with a holding-level SEIR model, we simulated the dynamic spread of an ASF-like pathogen introduced at M1514. This approach captured critical propagation dynamics, with epidemics spreading rapidly to 2% of municipalities within 100 days, confirming M1514’s role as a network propagation hub. Additionally, we demonstrated that early-stage projections exhibit significantly greater precision than later forecasts, providing more accurate estimates of both: final outbreak size and outbreak progression patterns. This enhanced early-stage predictive capacity creates a critical window for targeted interventions before uncertainty escalates and transmission processes become irreversibly established.

Epidemic simulations revealed limited network-wide dissemination, with only 10% of susceptible municipalities ultimately becoming infected. This moderate overall impact reflects the Austrian pig trade network’s inherent sparsity and hierarchical structure, which naturally limit extensive spread.^54^ These findings align with Avraam et al.,^58^ who demonstrated that network heterogeneity and community structure are key determinants of epidemic scale. The observed pattern of localized clusters interspersed with long-distance jumps reflects the multiscale nature of disease spread through complex trade systems.^59^ The propagation network exhibits a small-world structure^60^ characterized by densely connected clusters that enable localized transmission, while occasional long-distance jumps facilitate broader geographic spread. Collectively, these features highlight how network topology shapes epidemic outcomes across varying levels of trade activity.

Comparative analysis revealed fundamental differences in outbreak predictions between static and temporal network models.^44^^,^^61^^,^^62^ The static network overestimated the number of affected municipalities by 8.9 times by assuming all annual trade connections are simultaneously available, creating unlimited transmission potential.^63^ In reality, the temporal model showed that epidemics unfold sequentially: transmission requires trade events to occur in specific orders while infection is present. This demonstrates that accurate risk assessment requires explicit incorporation of trade chronologies.^61^

Following the initial introduction, the infection, if left uncontrolled, rapidly spreads within the vicinity of the seed municipality. This early phase is driven by intense holding-to-holding transmission, which precedes occasional long-range movements. These rare jumps subsequently seed new areas, where local to medium-range transmission further amplifies the outbreak. This localized transmission pattern reflects the homophilic nature of swine trade in Austria, where animal transfers predominantly occur between holdings located within the same federal state.^54^ In densely populated livestock regions, the close proximity between holdings facilitates rapid contagion, raising the risk of severe epidemics^64^ compared to more sparsely populated regions. Although stochastic local dispersion increases substantially epidemic size, it has a limited impact on the overall geographic extent of the outbreak.^57^

Notably, long-distance transmission events, though uncommon, were responsible for 20% of affected municipalities that would otherwise have remained uninfected. Therefore, mitigating these rare but critical jumps is essential to prevent the transition from localized outbreaks to nationwide epidemics. Long-distance trades can disproportionately alter disease dynamics by creating pathways that enable infections to spread across vast geographic areas, bypassing local containment efforts. This phenomenon, where infrequent events have outsized consequences, was described during FMD outbreaks in Great Britain,^55^ Uruguay,^4^ and Japan,^27^ where long-range animal movements were instrumental in seeding infections far from the original outbreak site, leading to widespread transmission. A similar pattern has been observed in human mobility studies, where long-distance travelers play a pivotal role in disseminating diseases across geographically disconnected regions.^65^ These findings highlight the dual role of epidemic dynamics: proximity-based spread determines the local case load, while occasional long-range jumps are primary drivers of geographical escalation.

Our results confirm that the risk of disease propagation is dynamic, fluctuating with trade activity.^62^^,^^66^ Specifically, the critical window for containment varies significantly depending on the timing of disease introduction. When the disease is introduced during a period of low trade activity (e.g., January),^54^ it spreads locally for approximately 40 days before the first long-distance infection jump occurs, with the probability of such an event remaining below 1%. In contrast, introduction during high-trade periods (e.g., April)^54^ shortens this critical window to 20 days, after which the probability of a long-distance jump rises to 11%. These findings define the drastically reduced operational window for effective containment efforts in high-trade seasons, making rapid detection and immediate response crucial to prevent wider spread. This disparity is explained by the underlying pattern of long-distance trades: while both January and April recorded 13 such events, outbreaks seeded in April coincided with the onset of the annual peak of long-distance trade events in May (29 events), resulting in a substantially higher probability of early transmission jumps. However, the observed multimodal peaks in infection jumps cannot be explained solely by trade intensity. Instead, we suggest it emerges from the complex interactions between multiple factors, including the timing at which infections reach key spreader nodes determined by network topology; non-trade transmission dynamics that amplify infection within high-density regions; and specific trade behaviors of individual holdings.

In both scenarios, the probability of a long-distance transmission jump (and subsequent spread to distant regions) increased substantially after 100 days. These time points (20, 40, and 100 days post-introduction) serve as easy-to-remember, critical stages for mitigation efforts. Delayed identification of infections or late reporting of laboratory test results allows the disease to spread unchecked, compromising containment efforts and increasing the risk of large-scale outbreaks.^27^^,^^67^^,^^68^^,^^69^ Such delays can also have severe consequences, not only for epidemic control but also for the economy, animal health, and welfare.^70^ Notably, if the outbreak extends beyond this critical window, control strategies must expand to target not only local areas but also distant regions affected by long-range trade movements.

Our results demonstrate that municipalities with high trade activity are not only more susceptible to disease incursion but also serve as key facilitators of further transmission. Among these, municipality M1491 emerged as a major driver of long-distance infection jumps, emphasizing its pivotal role in transmission dynamics (long-range spreader). These high-risk nodes represent critical targets for enhanced surveillance and strengthened biosecurity measures to mitigate the risk of widespread disease dissemination. This aligns with findings from other disease outbreaks, such as avian influenza and FMD, where disrupting trade hubs early in an outbreak has been shown to substantially reduce downstream infections.^71^^,^^72^^,^^73^^,^^74^ Similarly, studies in human epidemiology have demonstrated that targeted vaccination of highly connected individuals can disrupt transmission early in an outbreak, potentially halting the spread of infection more effectively.^75^

On a broader scale, our work reinforces the importance of international collaboration. Infectious diseases are not confined by political borders,^76^^,^^77^^,^^78^ and neither should efforts to control them. For Austria, the identification of high-risk entry points and the role of long-distance trade underscore an urgent need for sub-regional, cross-border working groups with key partners like Germany, the Czech Republic, and Slovenia. These groups should prioritize aligning pre-emptive surveillance in shared trade basins, developing joint simulation exercises, and creating protocols for reciprocal, real-time alerts for high-risk movements. This targeted cooperation is critical because European countries share interconnected, regulated^28^ animal trade networks, where vulnerabilities in one country can have ripple effects across the entire continent. Harmonized surveillance protocols, real-time cross-border data sharing, and coordinated response strategies are essential components of modern biosecurity frameworks.

In conclusion, Austria’s pig import activity increased the risk of exotic disease introduction, and the domestic trade network enables disease spread, posing a threat to animal health and food security. Therefore, early outbreak detection, prioritized surveillance in high-risk municipalities, and stringent biosecurity measures can significantly reduce disease spread. By targeting high-risk nodes and critical time windows (20/40/100 days), containment strategies can be optimized to effectively manage both localized and long-distance transmission dynamics. During periods of high trade activity, swift intervention is essential, as the disease can spread to distant regions twice as fast compared to introductions during low-trade periods. After 100 days, containment becomes more challenging due to the increased frequency of long-distance infection jumps, requiring strategies that target both local and distant geographical scales. Our network-based approach offers a scalable framework that can be applied to other regions and adapted for different swine diseases, enhancing overall preparedness, and resilience against emerging threats.

### Limitations of the study

The SEIR model used in this study assumes homogeneous mixing within and between compartments, which may not fully capture the complexities of real-world disease dynamics, such as variations in farm biosecurity practices and animal susceptibility (i.e., based on pig age categories). Moreover, the absence of biosecurity data for holdings may limit the model’s accuracy, as varying biosecurity levels significantly influence the holding’s vulnerability to infectious disease. Incorporating this information would enhance the model’s realism. Furthermore, the model uses fixed ASF-like parameters and therefore does not capture the diverse epidemiological traits of other swine pathogens or potential meteorological influences on transmission. The comparison of two introduction time points, representing seasonal extremes in trade activity, also limits the exploration of year-round dynamics. Finally, the analysis is based on historical data from 2021, which may not fully reflect future conditions due to evolving trade patterns. Nevertheless,^54^ demonstrated that the Austrian pig trade network has exhibited consistent annual cycles over seven years, suggesting some stability in trade dynamics over time.

Although the outbreak in our study was seeded in the municipality with the highest import activity, real-world introductions of foreign diseases may occur in unexpected locations, including regions with minimal import activity. Such scenarios emphasize the inherently unpredictable nature of disease incursions and underscore the need for adaptable surveillance and response strategies. Importantly, our framework remains applicable regardless of the initial entry point, as it enables the identification of likely transmission trajectories and the assessment of disease spread dynamics across the entire trade network. By applying this approach to any potential outbreak origin, authorities can proactively design targeted control measures that are both timely and spatially optimized to mitigate the risk of large-scale epidemics.

While the study focuses on disease spread through animal movements, it does not account for other potential transmission pathways like interactions with wild animals (e.g., wild boars and rats) or insect density, which can contribute to local transmission, especially for some swine diseases. Moreover, the trade network analyzed may not reflect the full complexity of actual trade dynamics, as informal trades or unreported movements (such as truck transits) could influence disease spread. Despite these limitations, the study offers valuable insights into how transboundary animal diseases could be introduced into Austria through high-import municipalities, providing a general overview of the vulnerabilities within the country’s swine industry and informing targeted surveillance and prevention strategies.

## Resource availability

### Lead contact

Requests for further information and resources should be directed to Gavrila Amadea Puspitarani (gavrila.puspitarani@vetmeduni.ac.at).

### Materials availability

This study did not generate new materials.

### Data and code availability

• Data: The raw data that supports the findings of this study are available from the Austrian Federal Ministry of Social Affairs, Health, Care and Consumer Protection (BMSGPK) but restrictions apply to the availability of these data, which were used under license for the current study, and so are not publicly available. Data are, however, available from the authors upon reasonable request and with permission of the data owner.
• Code: All original code has been deposited at Figshare: https://doi.org/10.6084/m9.figshare.29656082 and is publicly available as of the date of publication.
• Other item: Any additional information required to reanalyze the data reported in this paper is available from the lead contact upon request.

## Acknowledgments

The authors would like to thank the Austrian Federal Ministry of Social Affairs, Health, Care and Consumer Protection (BMSGPK) and the Austrian Agency for Health and Food Safety (AGES) for providing the data for this study. We thank Reinhard Fuchs for preparing the datasets, including data cleaning and pre-processing. We also extend our gratitude to Andrea Ladinig and Klemens Fuchs for their expert feedback and professional guidance. Finally, we are grateful to our colleagues at the Complexity Science Hub, Vienna, Austria, for their valuable brainstorming sessions and continuous support.

## Author contributions

Conceptualization, G.A.P. and A.D.-L.; methodology, G.A.P., H.S., and A.D.-L.; investigation, G.A.P. and H.S.; code writing, G.A.P., H.S., and E.C.; writing—original draft, G.A.P.; writing—review & editing, G.A.P., E.C., H.S., and A.D.-L; supervision, A.D.-L.

## Declaration of interests

The authors declare no competing interests.

## STAR★Methods

### Key resources table

**Table:** 

REAGENT or RESOURCE	SOURCE	IDENTIFIER
**Deposited data**		

Code availability	Original code used for the simulations and statistical to produce the results and figures of this article have been deposited on Figshare.	https://doi.org/10.6084/m9.figshare.29656082

**Software and algorithms**		

R software	R software (v.4.1.0)	https://www.R-project.org/
RStudio	RStudio Server “Juliet Rose” (v.1.4.1717)	http://www.rstudio.com/
Python programming language	IPython 8.25.0	https://doi.org/10.1109/MCSE.2007.53
Jupyter Notebook (version 7.2.0).	JupyterLab 4.2	https://doi.org/10.3233/978-1-61499-649-1-87
R package “sf” and “sp”	Spatial Data Science	https://r-spatial.org/book/
R package “rgeos”	Interface to Geometry Engine – Open Source (“GEOS”)	https://r-forge.r-project.org/projects/rgeos/
GeoPandas	geopandas	10.5281/zenodo.3946761
NetworkX	NetworkX	https://networkx.org/en/

### Method details

#### Pig mobility data

The dataset comprises origin-destination (OD) data, which captures daily live pig movements across Austria, 2015–2021, detailing transfers from a source holding to a destination holding (also called “flow data”). The dataset includes the date of movement, batch size, holding types, the number of animals per weight category in each holding, and the randomized 5 km-radius geo-coordinates (latitude and longitude, using the projected coordinate system EPSG 31287) of each holding. The data as well as the data cleaning processes have been previously detailed in Puspitarani et al.^54^ For the present study, only data from the year 2021 is used. We considered the following pig movement types: 1) *domestic*: movement of live pigs between holdings within Austria, 2) *slaughter*: domestic movement of live pigs intended for slaughter, 3) *abroad*: import or export of live pigs, and 4) *abroad-slaughter*: import of live pigs that are intended for slaughter.

The 2021 dataset included 23,722 pig holdings (e.g., farms, slaughterhouses, and trade centers) across 2,115 municipalities, with 250,137 movement records. In that year, 321 municipalities did not send any animals, and 198 did not receive any, resulting in 161 municipalities being inactive in terms of trade. The data included 145,104 slaughter movements, 100,327 domestic trades, 3,888 imports for slaughter, and 818 import/export movements. In 2021, Austria traded swine with 14 EU/EEA and two non-EU/EEA countries.

#### Administrative boundary data

The holdings’ geographical coordinates were overlaid over a shapefile of the Austrian municipalities^81^ using the GeoPandas library in Python.^82^ We extracted the name of the municipality where each holding was located using the function *st_GeoDataFrame*. To maintain confidentiality, all municipality names were anonymized in the analysis and reporting.

#### Networks of pig mobility

We constructed a 2021 static directed network at the holding level, where nodes represented the holding and edges represented a relationship (trade) between them. The directed graph *G* = (*V*, *E*) consisted of a set of nodes *V* representing holdings, and a set of directed edges *E*, representing the pig movement between them. Edges were weighted using the frequency of trade movements between holding pairs. To analyze the temporal dynamics, we also created a time-ordered sequence of daily snapshots, *G*_1_, …, *G*_365_, where each *G*_*t*_ represents the network for day *t*. The graphs were built using the NetworkX^83^ library with the DiGraph function, ensuring that networks were directed.

This network captures mobility flow, which describes interactions between entities based on movement patterns. While mobility studies often quantify interactions using the volume of moving entities (i.e., humans),^31^^,^^32^^,^^84^^,^^85^ our approach differs by using trade frequency between holding to define these connections.

#### Domestic mobility flow

Here, we aggregated the holding-level graph *G* to create a municipal-level graph *G*_*m*_. In this new static network, the nodes represent municipalities, and edges were defined based on the connections between their constituent holdings. To focus on domestic (i.e., occurring within Austria) movements between municipalities, we excluded import and export movements (i.e., “abroad” and “abroad-slaughter” types). For each municipality, we computed the outflow *F*_*out*_ and inflow *F*_*in*_ during 2021, which represented the total number of outgoing and incoming pig movements, respectively. In graph theory, these metrics are also referred to as out-strength and in-strength, respectively.^32^^,^^86^ The total flow (strength) was calculated as the sum of the in- and out-flow (*F*_*out*_ + *F*_*in*_).

To identify key municipalities in the network, we classified those with inflow in the upper quantile as high-risk for disease introduction (“sinks”). Conversely, we classified municipalities with outflow in the upper quantile as high-risk for disease spread (“amplifiers”). Municipalities with simultaneously high inflow and outflow were considered central to the network’s connectivity (“hubs”) and thus potentially critical for surveillance. We performed a Spearman correlation test to evaluate the rank-order relationship between inflow and outflow, determining whether municipalities with high outflow also exhibited high inflow (*p*-value ≤0.05 was considered significant).

#### Import flow and potential propagation network

To identify municipalities at higher risk of exotic disease introduction due to significant pig import activity, we filtered the dataset to include only import movements into Austria. For each municipality *m*, we calculated the number of import events using the following equation:

(Equation 1) $$F_{in,m}^{abroad}=\sum_{i=1}^{n}I\left({source}_{i}\notin \text{AT}\right)\cdot I\left({target}_{i}=m\right)$$

Where *I* identifies trades originating outside Austria (**AT**) and directed to municipality *m*. The municipality with the highest import flow was identified as the most probable entry point for the introduction of exotic diseases within Austria.

Further investigating the potential spread of exotic diseases from the municipality with the highest number of import trades, *m*∗, we extracted the static subgraph *G*′, referred to as the potential propagation network of *m*∗, which captures the connectivity between *m*∗ and other municipalities based on pig movements. Municipalities directly connected to *m*∗ were termed first-order neighbors (*k* = 1). The municipalities connected to these first-order neighbors were then classified as second-order neighbors (*k* = 2), and this process continued iteratively until no further connections could be identified (k-order neighbors). In this context, a “propagation step” corresponded to a single trade movement between two connected municipalities within *G*′, where step *k* corresponded to a trade between a municipality of order *k* − 1 and one of order *k*. The set of nodes that could be reached from *m*∗ within *k* steps was denoted as follows: $N_{k}\left(m^{*}\right)=\left\{j|A_{m^{*}j}^{k}>0\right\}$, where $A_{m^{*}j}^{k}>0$ indicates that there is a connection from *m*∗ to node *j* within *k* steps. This analysis aimed to identify municipalities at risk of infection following an incursion in the highest-import municipality and explore how diseases might propagate through the static network.

We examined the spatial characteristics of the potential propagation network *G*′ to assess whether trades predominantly occur over long or short distances and to evaluate how spatial proximity influences disease spread. For this purpose, we calculated the Euclidean distances between the geographic centroids of connected municipalities. The centroid *C*_*m*_ of a municipality *m* was determined from the shapefile of Austrian administrative boundaries.^81^ Then we calculated the Euclidean distances, *D*(*i*, *j*), between the geographic centroids of two connected municipalities *i* and *j*.

We analyzed the distribution of the Euclidean trade distances (in km) across different steps in the propagation network. For each step *k*, we calculated the average distance ${\bar{D}}_{k}$ over all trades occurring at k-order from *m*∗ and examined how these distances varied across steps (orders). Furthermore, all Euclidean distances between connected municipalities in the potential propagation network were calculated, providing a statistical summary of these trade distances. In this work, we defined long-distance threshold as any trade with a Euclidean distance greater than two standard deviations above the mean of all trade distances (this same threshold was applied in the transmission model to identify long-distance infection jumps). We then quantified the frequency of these long-distance events for each month of the study period.

#### Epidemic model

Here, we used the temporal subgraphs *G*_*t*_ (unlike the static network used in previous analyses) to simulate disease transmission over time, capturing the dynamic nature of disease spread. We developed a holding-level compartmental contagion model that incorporated two transmission pathways: (1) direct contact via trade (e.g., introduction of infected pigs) and (2) indirect short-distance transmission (e.g., via fomites or arthropod vectors). This approach allowed for a more realistic representation of disease propagation dynamics between holdings.

Additionally, we modeled infection dynamics at the farm level.

##### Transmission dynamic within holding

We used a stochastic Susceptible-Exposed-Infectious-Removed *SEIR* model to capture the transmission dynamics within each holding, assuming a homogeneous mixing of pigs. The model compartmentalized the farm population into four categories: pigs that are susceptible to the disease *S*, pigs that have been exposed to the disease *E*, i.e., infected but not yet contagious, pigs that are infectious *I*, and pigs that have been removed *R*, i.e., dead.

The total pig population for each holding was based on the annual census. We assumed herd sizes remained constant during the study period, ensuring sufficient pigs were available for trade between holdings. Therefore, the *SEIR* model excluded natural birth and death rates (except deaths related to infections). At *t* = 0, all pigs in every holding were considered susceptible.

Within a herd, if a susceptible pig came into contact with an infectious pig, it became exposed with probability *P*, following a frequency-dependent transmission with a PERT distribution:

(Equation 2) $$P_{i}\left(t\right)=1-e^{-\beta I_{i}\left(t\right)/N_{i}\left(t\right)}$$

Where *β* is the transmission rate, *I*_*i*_(*t*) represents the number of infectious pigs at time *t*, and *N*_*i*_(*t*) = *S*_*i*_(*t*) + *E*_*i*_(*t*) + *I*_*i*_(*t*) is the total number of pigs in herd *i* at time *t*. After the latent period *μ*, pigs transitioned from *E* to the infectious state *I*. Subsequently, the pigs were removed when the infectious period *σ* ends. We assumed a disease with 100% lethality, so that all pigs were removed at the end of the infectious period. The epidemiological parameters were based on those of an ASF-like virus, which is not currently present in Austria, serving as an example of a potential exotic disease that could be introduced through the importation of live animals. The schematic representation of the transition between compartments and the parameter values is provided in Figure S1 and Table 1 respectively. This parameterization provides a concrete case study for an exotic ASF-like threat. However, our analytical framework is readily adaptable, allowing for the investigation of other swine pathogens with different epidemiological characteristics.

**Table 1:** Parameters used in the epidemiological model

Parameter	Definition	Value
*β*	Average transmission rate from an infectious pig to a susceptible one	pert(0.2; 0.4; 0.6) *day*^−1^^46^^,^^47^
*μ*	Average duration of the latent period in pigs	pert(3; 4; 5) days^46^^,^^47^
*σ*	Average duration of the infectious period in pigs	pert(3; 7; 14) days^46^^,^^47^
*D*	Spatial range of the local transmission kernel	5 km^79^^*a*^.
Prevalence at *t* = 1	Proportion of infected pigs in the seeded herd at *t* = 1	10%
*β*^*loc*^	Maximum local transmission rate	0.0001 *day*^s−1^^80^

This value represents a conservative estimate for capturing local transmission events.

Our model utilizes fixed epidemiological parameters to isolate the specific effects of trade network structure and timing on disease spread. This simplifying assumption allows us to clearly attribute simulated outbreak dynamics to the movement data, providing a foundational understanding of network-driven risks without the confounding influence of seasonal transmission effects.

##### Transmission dynamics between holdings

At the start of each simulation, *t* = 0, all nodes in the network were disease-free. The outbreak was initiated in a holding located in the municipality with the highest imports, *m*∗, where one holding was randomly selected as the initial infection source (seed). In the selected holding, 10% of the susceptible pigs were infected by *t* = 1, representing 10% of the total herd size. A holding was considered to be infected if at least one pig from the *E* or *I* compartment was present.

When pigs were transferred from holding *i* to *j*, they were drawn randomly from *i*'s compartments (*S*, *I*, or *E*), based on the recorded number of transferred animals. These pigs were then assigned to their corresponding compartments in holding *j*. The introduction of at least one pig from *E* or *I* compartment caused the susceptible holding *j* to become infected. The localized contagion process was modeled using a Gaussian transmission kernel with a 5-km radius.^79^^,^^80^ This value represents a conservative estimate for capturing high-risk local transmission events, and was also selected to align with international disease control guidelines, falling between the 3-km Protection Zone and 10-km Surveillance Zone mandated by the World Organization of Animal Health (WOAH) and EU legislation for diseases like ASF.^26^^,^^28^ In this model, the probability of transmission *P*_*loc*_ decreases as the distance between the infected holding *i* and the susceptible holding *j* increases, defined as:

(Equation 3) $$P_{j}^{loc}\left(t\right)=1-e^{-\beta^{loc}d_{\left(i,j\right)}I_{i}\left(t\right)}$$

Where, for each time step *t*, *β*^*loc*^*d*_(*i*,*j*)_ is the distance-dependent transmission rate and *d*_(*i*,*j*)_ is the distance between the infected holding *i* and the susceptible holding *j*. The force of infection is proportional to the number of infected pigs *I*_*i*_ in the infected holding *i*.

The model was executed over 1,000 iterations. Each simulation ran for 365 days, starting on January 1, 2021 (*t* = 1) and ending on December 31, 2021 (*t* = 365).

Additionally, a comparative analysis was conducted to assess the impact of the timing of disease introduction on infection dynamics. Specifically, we simulated the incursion of an exotic disease in April, a period of high trade activity in Austria,^54^ and compared the resulting epidemic trajectory to that of an introduction in January, a period of low trade activity. In this scenario, each simulation started on a randomly selected day in April and ended on December 31, 2021.

#### Epidemic size

We evaluated the epidemic size of an exotic disease introduced via trade in Austria by calculating the total number of infected and removed pigs (*E* + *I* + *R*) and infected holdings (*E* + *I*) at each time step in the propagation network *G*′. Additionally, we provided a breakdown of infected pigs, *E* + *I*, which reflects the persistence of the disease in the population, while *R* reflects the disease burden in terms of mortality. To summarize the spread across holdings, we reported the maximum, minimum, median, and 95% confidence intervals of infected holdings at each time step.

To estimate the number of infected municipalities, we determined the count of unique municipalities reached by infection in each simulation iteration. Using a bootstrapping approach, we generated 1,000 resamples by randomly sampling epidemic sizes with replacement. The mean and 95% confidence interval (CI) of these resampled values were then calculated and visualized in a histogram.

We compared the epidemic size of an ASF-like disease introduced in January versus April by analyzing the first 30 days of the outbreak. This time frame reflects a realistic scenario in which the disease would no longer circulate unnoticed or undetected, as notification would have occurred. Additionally, it accounts for the period during which the outbreak may not yet be fully contained.^69^^,^^87^^,^^88^^,^^89^

#### Municipality-level vulnerability to infection

We calculated the likelihood of infection for each municipality by determining how frequently it was affected across all iterations. For each iteration, we recorded whether a municipality was reached by the infection and then counted these occurrences. The frequencies were normalized by the total number of iterations, producing likelihood values ranging from 0 (never reached) to 1 (always reached).

#### Analysis of long-distance infection jumps

For each pair of municipalities where a successful infection occurred in the propagation network, we calculated the distance (in km) between their centroids. Any infection event exceeding the predefined long-distance threshold was classified as a long-distance infection.

These jumps were analyzed to determine their frequency and normalized likelihood across all simulation iterations. The likelihood of long-distance jumps between municipalities was computed as the number of occurrences divided by the total number of iterations.

If a successful long-distance infection occurred during an iteration, it was marked as present; otherwise, it was marked as absent. We calculated the duration of the initial localized stage (until the first long-distance jump) per simulation, with means and 95% CIs derived via bootstrap resampling. To quantify the relative contribution of the transmission route, we performed a connected component analysis on the propagation network *G*′ after removing all long-distance edges. The proportion of affected nodes attributable to each transmission pathway was calculated by classifying those within the seed’s connected component as local spread, and all others as long-distance initiated.

We visualized successful long-distance infection jumps using the propagation network *G*′, where edge weights represent the likelihood of infection jump occurrence, therefore creating a “long-distance infection propagation network”. For each node (municipality), we computed the in- and out-strength centrality by summing the weights of incoming and outgoing edges, respectively. In this context, in-strength centrality represents the likelihood of a municipality being infected through incoming long-distance trades, while out-strength centrality represents the likelihood of a municipality spreading infection to others through outgoing long-distance trades.

Additionally, we analyzed the timing of long-distance infection jumps to identify key patterns. A histogram with a kernel density estimation (KDE) highlighted periods with the highest concentration of jumps. To determine if the temporal pattern of long-distance jumps was driven by underlying trade activity, we fitted a Poisson regression model, with the weekly count of infection jumps regressed on the weekly volume of all trades. Finally, a heatmap visualized the temporal and spatial distribution of these jumps, identifying specific time frames when certain municipalities played a major role in disease spread.

### Quantification and statistical analysis

Computational modeling and data processing were performed using R software (v.4.1.0)^90^ within RStudio Server “Juliet Rose” (v.1.4.1717)^91^ and Python (8.25.0)^92^ via Jupyter Notebook (version 7.2.0).^93^ Spatial data management and overlay of holding coordinates onto administrative boundaries were conducted using the GeoPandas library^82^ in Python and the “sp”, “sf”,^94^ “rgeos”^95^ package in R. Network constructions and topology analyses utilized NetworkX library.^83^ Data visualizations, including kernel density estimations (KDE) to identify peaks in infection risks, were also generated in Python. All original code used for the simulations and statistical evaluations has been deposited at Figshare (https://doi.org/10.6084/m9.figshare.29656082).

The study utilized several statistical tests. A Spearman correlation test was conducted to determine the relationship between municipal-level trade inflow and outflow, with a *p*-value ≤0.05 considered significant. To compare the epidemic trajectories of the January and April introduction scenarios, Mood’s median test was applied to the number of holdings and pigs in the exposed and infectious compartments. Poisson regression model was fitted to analyze the association between weekly trade volumes and the frequency of long-distance infection jumps. For spatial analysis, a connected component analysis was used to distinguish between municipalities affected by local spread versus those seeded by long-distance events.

The sample size (*n*) in this study primarily refers to the 1,000 stochastic simulation iterations performed for each seeding scenario to capture the variability of disease spread. Centrality in the data are reported as either the mean (e.g., for trade distances and municipal infections) or the median (e.g., for temporal dynamics of infected holdings). Precision and dispersion are represented by standard deviation (SD) and 95% confidence intervals (CI). These CIs were derived using a bootstrap resampling approach with 1,000 iterations to provide robust estimates of epidemic size and the duration of the initial local spread phase.

Specific statistical details regarding the experiments can be found throughout the manuscript and supplemental files. The epidemiological parameters and the PERT distributions used for stochastic modeling are consolidated in Table 1. Finally, extended comparisons between seasonal seeding times and the associated statistical distributions are provided in Data S1.

## References

[1] Fèvre E.M., Bronsvoort B.M.d.C., Hamilton K.A., Cleaveland S., Cleaveland S. (2006). Animal movements and the spread of infectious diseases. Trends Microbiol..

[2] Roche X., Rozstalnyy A., TagoPacheco D., Pittiglio C., Kamata A., Alcrudo D.B., Bisht K., Karki S., Kayamori J., Larfaoui F. (2020).

[3] Shanafelt D.W., Perrings C.A. (2017). Foot and mouth disease: the risks of the international trade in live animals. Rev. Sci. Tech..

[4] Iriarte M.V., Gonzáles J.L., Gil A.D., de Jong M.C.M. (2023). Animal movements and fmdv transmission during the high-risk period of the 2001 fmd epidemic in uruguay. Transbound. Emerg. Dis..

[5] Bouma A., Elbers A.R.W., Dekker A., de Koeijer A., Bartels C., Vellema P., van der Wal P., van Rooij E.M.A., Pluimers F.H., de Jong M.C.M. (2003). The foot-and-mouth disease epidemic in the netherlands in 2001. Prev. Vet. Med..

[6] Agriculture and Rural Economy Directorate. Livestock identification and traceability: guidance (2021). https://www.gov.scot/publications/livestock-identification-and-traceability-guidance/pages/cattle/.

[7] Commission Regulation (EU) (2022). Regulation - 2022/175 - en - eur-lex. https://eur-lex.europa.eu/legal-content/EN/TXT/?uri=CELEX%3A32022R0175.

[8] Puspitarani G.A., Liao Y.-S.J., Fuchs R., Desvars-Larrive A. (2025). Investigating the impact of edge weight selection on the pig trade network topology. Epidemics.

[9] You S., Liu T., Zhang M., Zhao X., Dong Y., Wu B., Wang Y., Li J., Wei X., Shi B. (2021). African swine fever outbreaks in china led to gross domestic product and economic losses. Nat. Food.

[10] Schulz L.L., Tonsor G.T. (2015). Assessment of the economic impacts of porcine epidemic diarrhea virus in the united states. J. Anim. Sci..

[11] Ganges L., Crooke H.R., Bohórquez J.A., Postel A., Sakoda Y., Becher P., Ruggli N. (2020). Classical swine fever virus: the past, present and future. Virus Res..

[12] Friedrich-Loeffler-Institut. Karten zur asp (2025). https://www.fli.de/de/aktuelles/tierseuchengeschehen/afrikanische-schweinepest/karten-zur-afrikanischen-schweinepest/.

[13] Frössling J., Ohlson A., Björkman C., Håkansson N., Nöremark M. (2012). Application of network analysis parameters in risk-based surveillance – examples based on cattle trade data and bovine infections in sweden. Prev. Vet. Med..

[14] Tobias I.G., Rojo B., Talha, Filip S., Ingrid T.J.H., Masood B. (2021). Topological Methods in Data Analysis and Visualization VI.

[15] Kao R.R., Danon L., Green D.M., Kiss I.Z. (2006). Demographic structure and pathogen dynamics on the network of livestock movements in great britain. Proc. Biol. Sci..

[16] Marcos, Eraldo R., Sima M.R.F., Federico F.,M.J., Ronaldo M., Sofia M.G.T.A., Botta (2023). Complex Networks XIV.

[17] Gilbert M., Mitchell A., Bourn D., Mawdsley J., Clifton-Hadley R., Wint W. (2005). Cattle movements and bovine tuberculosis in great britain. Nature.

[18] Mellor P.S., Kitching R.P., Wilkinson P.J. (1987). Mechanical transmission of capripox virus and african swine fever virus by stomoxys calcitrans. Res. Vet. Sci..

[19] Olesen A.S., Hansen M.F., Rasmussen T.B., Belsham G.J., Bødker R., Bøtner A. (2018). Survival and localization of african swine fever virus in stable flies (stomoxys calcitrans) after feeding on viremic blood using a membrane feeder. Vet. Microbiol..

[20] Ribbens S., Dewulf J., Koenen F., Maes D., de Kruif A. (2007). Evidence of indirect transmission of classical swine fever virus through contacts with people. Vet. Rec..

[21] Sanson R.L., Struthers G., King P., Weston J.F., Morris R.S. (1993). The potential extent of transmission of foot-and-mouth disease: a study of the movement of animals and materials in southland, new zealand. N. Z. Vet. J..

[22] Mazur-Panasiuk N., Woźniakowski G. (2020). Natural inactivation of african swine fever virus in tissues: Influence of temperature and environmental conditions on virus survival. Vet. Microbiol..

[23] Nuanualsuwan S., Songkasupa T., Boonpornprasert P., Suwankitwat N., Lohlamoh W., Nuengjamnong C. (2022). Persistence of african swine fever virus on porous and non-porous fomites at environmental temperatures. Porcine Health Manag..

[24] Guinat C., Reis A.L., Netherton C.L., Goatley L., Pfeiffer D.U., Dixon L. (2014). Dynamics of african swine fever virus shedding and excretion in domestic pigs infected by intramuscular inoculation and contact transmission. Vet. Res..

[25] Olesen A.S., Lohse L., Boklund A., Halasa T., Gallardo C., Pejsak Z., Belsham G.J., Rasmussen T.B., Bøtner A. (2017). Transmission of african swine fever virus from infected pigs by direct contact and aerosol routes. Vet. Microbiol..

[26] World Organisation for Animal Health (2019). African swine fever: Aetiology epidemiology diagnosis prevention and control references. https://www.woah.org/en/document/african_swine_fever/.

[27] Hayama Y., Firestone S.M., Stevenson M.A., Yamamoto T., Nishi T., Shimizu Y., Tsutsui T. (2019). Reconstructing a transmission network and identifying risk factors of secondary transmissions in the 2010 foot-and-mouth disease outbreak in japan. Transbound. Emerg. Dis..

[28] European Parliament and The Council (2016). Regulation (EU) 2016/429. https://eur-lex.europa.eu/eli/reg/2016/429/oj.

[29] Tildesley M.J., Brand S., Brooks Pollock E., Bradbury N.V., Werkman M., Keeling M.J. (2019). The role of movement restrictions in limiting the economic impact of livestock infections. Nat. Sustain..

[30] Thulke H.H., Eisinger D., Beer M. (2011). The role of movement restrictions and pre-emptive destruction in the emergency control strategy against csf outbreaks in domestic pigs. Prev. Vet. Med..

[31] Balcan D., Colizza V., Gonçalves B., Hu H., Ramasco J.J., Vespignani A. (2009). Multiscale mobility networks and the spatial spreading of infectious diseases. Proc. Natl. Acad. Sci. USA.

[32] Rong C., Ding J., Li Y. (2025). An Interdisciplinary Survey on Origin-destination Flows Modeling: Theory and Techniques. ACM Comput. Surv..

[33] Mazzoli M., Molas A., Bassolas A., Lenormand M., Colet P., Ramasco J.J. (2019). Field theory for recurrent mobility. Nat. Commun..

[34] Yang H., Li M., Guo B., Zhang F., Wang P. (2023). A vector field approach for identifying anomalous human mobility. IET Intell. Transp. Syst..

[35] Pastor-Escuredo D., Frias-Martinez E. (2020). Flow descriptors of human mobility networks. arXiv.

[36] Anderson R.M., Jackson H.C., May R.M., Smith A.M. (1981). Population dynamics of fox rabies in europe. Nature.

[37] Keeling M.J., Rohani P. (2011).

[38] Ferguson N.M., Donnelly C.A., Anderson R.M. (2001). Transmission intensity and impact of control policies on the foot and mouth epidemic in great britain. Nature.

[39] Kao R.R. (2002). The role of mathematical modelling in the control of the 2001 fmd epidemic in the uk. Trends Microbiol..

[40] Lee H.S., Thakur K.K., Bui V.N., Bui A.N., Dang M.V., Wieland B. (2019). Simulation of control scenarios of porcine reproductive and respiratory syndrome in nghe an province in vietnam. Transbound. Emerg. Dis..

[41] Thakur K.K., Revie C.W., Hurnik D., Poljak Z., Sanchez J. (2015). Simulation of between-farm transmission of porcine reproductive and respiratory syndrome virus in ontario, canada using the north american animal disease spread model. Prev. Vet. Med..

[42] Hasahya E., Thakur K.K., Dione M.M., Wieland B., Oba P., Kungu J., Lee H.S. (2021). Modeling the spread of porcine reproductive and respiratory syndrome among pig farms in lira district of northern uganda. Front. Vet. Sci..

[43] Yadav S., Widmar N.J.O., Weng H.-Y. (2016). Modeling classical swine fever outbreak-related outcomes. Front. Vet. Sci..

[44] Acosta A., Cardenas N.C., Imbacuan C., Lentz H.H.K., Dietze K., Amaku M., Burbano A., Gonçalves V.S.P., Ferreira F. (2022). Modelling control strategies against classical swine fever: Influence of traders and markets using statautstatic and temporal networks in ecuador. Prev. Vet. Med..

[45] Martínez-López B., Ivorra B., Ramos A.M., Sánchez-Vizcaíno J.M. (2011). A novel spatial and stochastic model to evaluate the within- and between-farm transmission of classical swine fever virus. i. general concepts and description of the model. Vet. Microbiol..

[46] Vergne T., Andraud M., Bonnet S., De Regge N., Desquesnes M., Fite J., Etore F., Garigliany M.M., Jori F., Lempereur L. (2021). Mechanical transmission of african swine fever virus by *stomoxys calcitrans* : Insights from a mechanistic model. Transbound. Emerg. Dis..

[47] Guinat C., Gogin A., Blome S., Keil G., Pollin R., Pfeiffer D.U., Dixon L. (2016). Transmission routes of african swine fever virus to domestic pigs: current knowledge and future research directions. Vet. Rec..

[48] Machado G., Vilalta C., Recamonde-Mendoza M., Corzo C., Torremorell M., Perez A., VanderWaal K. (2019). Identifying outbreaks of porcine epidemic diarrhea virus through animal movements and spatial neighborhoods. Sci. Rep..

[49] Sethi S.P. (2021).

[50] Li L., Zheng N., Liu C., Wang Z., Jin Z. (2025). Optimal control of vaccination for an epidemic model with standard incidence rate. J. Theor. Biol..

[51] Sun G.-Q., He R., Hou L.-F., Luo X., Gao S., Chang L., Wang Y., Zhang Z.-K. (2025). Optimal control of spatial diseases spreading in networked reaction–diffusion systems. Phys. Rep..

[52] Shi S., Wang Z., Chen X., Fu F. (2024). Determinants of successful disease control through voluntary quarantine dynamics on social networks. Math. Biosci..

[53] Dubé, C., Garner, G., Stevenson, M., Sanson, R., Estrada, C., and Willeberg, P. (2007). The use of epidemiological models for the management of animal diseases. In OIE Conference ((13–23). https://www.woah.org/app/uploads/2021/03/2007-013-023-dube-a.pdf.

[54] Puspitarani G.A., Fuchs R., Fuchs K., Ladinig A., Desvars-Larrive A. (2023). Network analysis of pig movement data as an epidemiological tool: an Austrian case study. Sci. Rep..

[55] Gibbens J.C., Sharpe C.E., Wilesmith J.W., Mansley L.M., Michalopoulou E., Ryan J.B., Hudson M. (2001). Descriptive epidemiology of the 2001 foot-and-mouth disease epidemic in Great Britain: the first five months. Vet. Rec..

[56] Ekwem D., Enright J., Hopcraft J.G.C., Buza J., Shirima G., Shand M., Mwajombe J.K., Bett B., Reeve R., Lembo T. (2023). Local and wide-scale livestock movement networks inform disease control strategies in East Africa. Sci. Rep..

[57] Green D.M., Kiss I.Z., Kao R.R. (2006). Modelling the initial spread of foot-and-mouth disease through animal movements. Proc. Biol. Sci..

[58] Avraam D., Hadjichrysanthou C. (2025). The impact of contact-network structure on important epidemiological quantities of infectious disease transmission and the identification of the extremes. J. Theor. Biol..

[59] Xian J., Liu M., Cheng X., Yang M., Xie T., Wang X., Liu M., Zhang Y.-C., Yang D., Sun G.-Q., Ye J. (2025). Modelling multiscale infectious disease in complex systems. Phys. Rep..

[60] Watts D.J., Strogatz S.H. (1998). Collective dynamics of ‘small-world’ networks. Nature.

[61] Cardenas N.C., VanderWaal K., Veloso F.P., Galvis J.O.A., Amaku M., Grisi-Filho J.H.H. (2021). Spatio-temporal network analysis of pig trade to inform the design of risk-based disease surveillance. Prev. Vet. Med..

[62] Lentz H.H.K., Koher A., Hövel P., Gethmann J., Sauter-Louis C., Selhorst T., Conraths F.J. (2016). Disease spread through animal movements: A static and temporal network analysis of pig trade in Germany. PLoS One.

[63] Holme P., Saramäki J. (2012). Temporal networks. Phys. Rep..

[64] Backer J.A., Hagenaars T.J., Nodelijk G., van Roermund H.J.W. (2012). Vaccination against foot-and-mouth disease i: Epidemiological consequences. Prev. Vet. Med..

[65] Lessani M.N., Li Z., Jing F., Qiao S., Zhang J., Olatosi B., Li X. (2024). Human mobility and the infectious disease transmission: a systematic review. Geo-Spatial Inf. Sci..

[66] Aklilu, Y. Livestock trade in Karamoja, Uganda: An update of market dynamics and trends (2017). URL: www.karamojaresilience.org.

[67] Benítez H., Cornes F., Dorso C., Frank G. (2024). Disease spreading through complex small world networks. Med. Res. Arch..

[68] Costard S., Zagmutt F.J., Porphyre T., Pfeiffer D.U. (2015). Small-scale pig farmers’ behavior, silent release of african swine fever virus and consequences for disease spread. Sci. Rep..

[69] Eschbaumer M., Vögtlin A., Paton D.J., Barnabei J.L., Sanchez-Vazquez M.J., Pituco E.M., Rivera A.M., O’Brien D., Nfon C., Brocchi E. (2020). Non-discriminatory exclusion testing as a tool for the early detection of foot-and-mouth disease incursions. Front. Vet. Sci..

[70] Carpenter T.E., O’Brien J.M., Hagerman A.D., Mccarl B.A. (2011). Epidemic and economic impacts of delayed detection of foot-and-mouth disease: A case study of a simulated outbreak in California. J. Vet. Diagn. Invest..

[71] Dyck J., Harvey D., Blayney D. (2006). Economic effects of animal diseases linked to trade dependency. https://www.ers.usda.gov/amber-waves/2006/april/economic-effects-of-animal-diseases-linked-to-trade-dependency?utm_source=chatgpt.com.

[72] Cardenas N.C., Sykes A.L., Lopes F.P.N., Machado G. (2022). Multiple species animal movements: network properties, disease dynamics and the impact of targeted control actions. Vet. Res..

[73] Kinsley A.C., Perez A.M., Craft M.E., Vanderwaal K.L. (2019). Characterization of swine movements in the United States and implications for disease control. Prev. Vet. Med..

[74] Farmer N.H. (2021). Practical use of pig movement data for disease interventions. https://www.nationalhogfarmer.com/hog-health/practical-use-of-pig-movement-data-for-disease-interventions?utm_source=chatgpt.com.

[75] Gulden T.R., Hartnett G.S., Vardavas R., Kravitz D. (2021).

[76] Food and Agriculture Organization of the United Nations (2018). Outbreak of African swine fever threatens to spread from China to other Asian countries. https://www.fao.org/newsroom/detail/Outbreak-of-African-swine-fever-threatens-to-spread-from-China-to-other-Asian-countries/es.

[77] de Bengy Puyvallée A., Kittelsen S. (2019).

[78] Mur L., Martínez-López B., Sánchez-Vizcaíno J.M. (2012). Risk of african swine fever introduction into the European Union through transport-associated routes: returning trucks and waste from international ships and planes. BMC Vet. Res..

[79] VanderWaal K., Enns E.A., Picasso C., Alvarez J., Perez A., Fernandez F., Gil A., Craft M., Wells S. (2017). Optimal surveillance strategies for bovine tuberculosis in a low-prevalence country. Sci. Rep..

[80] Andraud M., Hammami P., Hayes B.H., Galvis J.A., Vergne T., Machado G., Rose N. (2022). Modelling african swine fever virus spread in pigs using time-respective network data: Scientific support for decision makers. Transbound. Emerg. Dis..

[81] Statistik Austria (2019). Gliederung Österreichs in Gemeinden - Dataset - data.gv.at. https://www.data.gv.at/katalog/en/dataset/stat_gliederung-osterreichs-in-gemeinden14f53.

[82] Jordahl, K., den Bossche, J. V., Fleischmann, M., Wasserman, J., McBride, J., Gerard, J., Tratner, J., Perry, M., Badaracco, A. G., Farmer, C., et al. geopandas/geopandas: v0.8.1 (2020). doi:10.5281/zenodo.3946761.

[83] Hagberg A.A., Schult D.A., Swart P.J., Varoquaux G., Vaught T., Millman J. (2008). Proceedings of the 7th Python in Science Conference (SciPy2008). Pasadena, CA, USA.

[84] Wesolowski A., Eagle N., Tatem A.J., Smith D.L., Noor A.M., Snow R.W., Buckee C.O. (2012). Quantifying the impact of human mobility on malaria. Science.

[85] Jia J.S., Lu X., Yuan Y., Xu G., Jia J., Christakis N.A. (2020). Population flow drives spatio-temporal distribution of covid-19 in China. Nature.

[86] Wasserman S., Faust K. (1994).

[87] National Audit Office (2002). https://www.nao.org.uk/wp-content/uploads/2002/06/0102939.pdf.

[88] Ferguson N.M., Donnelly C.A., Anderson R.M. (2001). The foot-and-mouth epidemic in great britain: pattern of spread and impact of interventions. Science.

[89] Tago D., Hammitt J.K., Thomas A., Raboisson D. (2016). The impact of farmers’ strategic behavior on the spread of animal infectious diseases. PLoS One.

[90] R Core Team (2021). https://www.R-project.org/.

[91] RStudio Team (2020). http://www.rstudio.com/.

[92] Perez F., Granger B.E. (2007). Ipython: A system for interactive scientific computing. Comput. Sci. Eng..

[93] Kluyver T., Ragan-Kelley B., Pérez F., Granger B., Bussonnier M., Frederic J., Kelley K., Hamrick J., Grout J., Corlay S. (2016).

[94] Pebesma E., Bivand R. (2023). https://r-spatial.org/book/.

[95] Bivand, R., and Rundel, C. rgeos: Interface to Geometry Engine - Open Source (‘GEOS’) (2023). https://r-forge.r-project.org/projects/rgeos/(or) https://libgeos.org (or) http://rgeos.r-forge.r-project.org/index.html

