# Supplementary material for: Risk of exotic disease introduction and propagation in the Austrian swine trade network
Source: iScience. 2026 Feb 2;29(3):114868. doi: 10.1016/j.isci.2026.114868 (PMC12925288; doi:10.1016/j.isci.2026.114868)
Supplement: Data S1. Comparison of epidemic dynamics at different seeding times in a PDF [file mmc2.pdf]

Supplementary Data 1: Epidemic dynamics and impact of a hypothetical introduction of an ASF-like disease in Austria during a high trade season (April)

This supplementary document examines the impact of seeding time on epidemic dynamics. April was selected based on trade seasonality data, which indicates a period of heightened network activity in Austria[1]. In contrast, January represents a period of low trade activity (January scenario is presented in the main text).

In the April simulation, the disease was introduced on a randomly selected date within April, with the initial infection occurring in a randomly selected holding within m^* (M1514, the highest-import municipality).

Epidemic dynamics and impact

We reported the first 30 days of disease spread after the hypothetical introduction of an ASF-like disease. The median introduction time was at day 106 (95%CI:[105.8 - 106.9]). Variably across simulation was minimal, as indicated by the narrow 95% confidence interval (CI), with a coefficient of variation (CV) of 0.16 for exposed compartment E and 0.21 for infectious compartment I (Figure 3).

After 30 days, the number of infected holdings reached 12 (95%CI: [8 - 15.2]), representing approximately 0.05% (95%CI: [0.03 – 0.06]) of all trading holdings in Austria in 2021, with a CV of 0.13 (main text Figure 4).

Weekly growth rates of infected holdings corroborated the presence of distinct epidemic phases. Between days 113 - 119, the number of infected holdings surged by 300%, indicating rapid early transmission. Growth then decelerated to an average of 67-23% between days 120 - 133, followed by a further decline to -54% between days 132-135.

At the municipality level, bootstrapping analysis indicated that an average of 8 (95% CI: [7.7 - 8.2]) municipalities (0.4%) were affected.

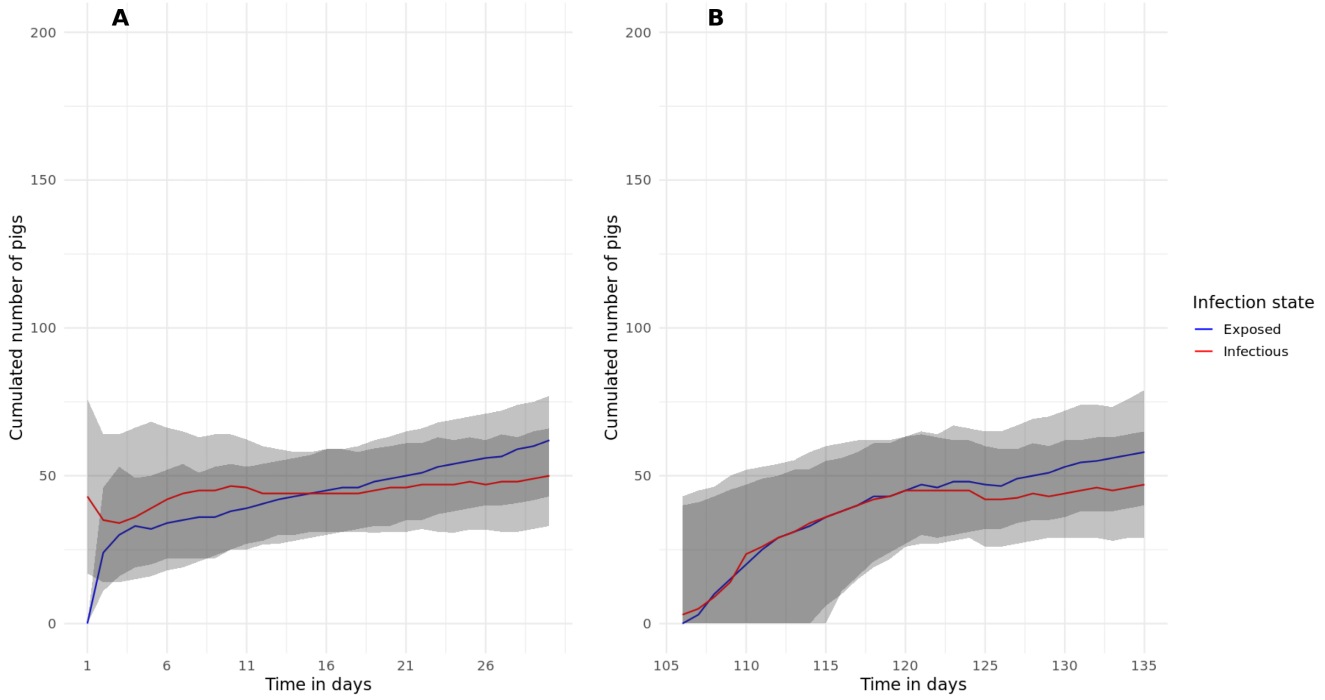

Figure 1: **Temporal dynamics of exposed E and infectious I pigs in a 30-day simulation after hypothetical introduction of an ASF-like disease in Austria on (A) 1st of January and (B) April 2021.** One holding, located in the municipality M1514 (the highest-import municipality), was randomly selected as the origin of the outbreak. The blue and red lines represent the cumulative median counts of exposed E and infectious I pigs, respectively. Shaded gray areas indicate the 95% confidence intervals (CI), reflecting variability across 1,000 simulations. The wider CIs at later time points highlight increased uncertainty in the model's predictions as the epidemic progresses.

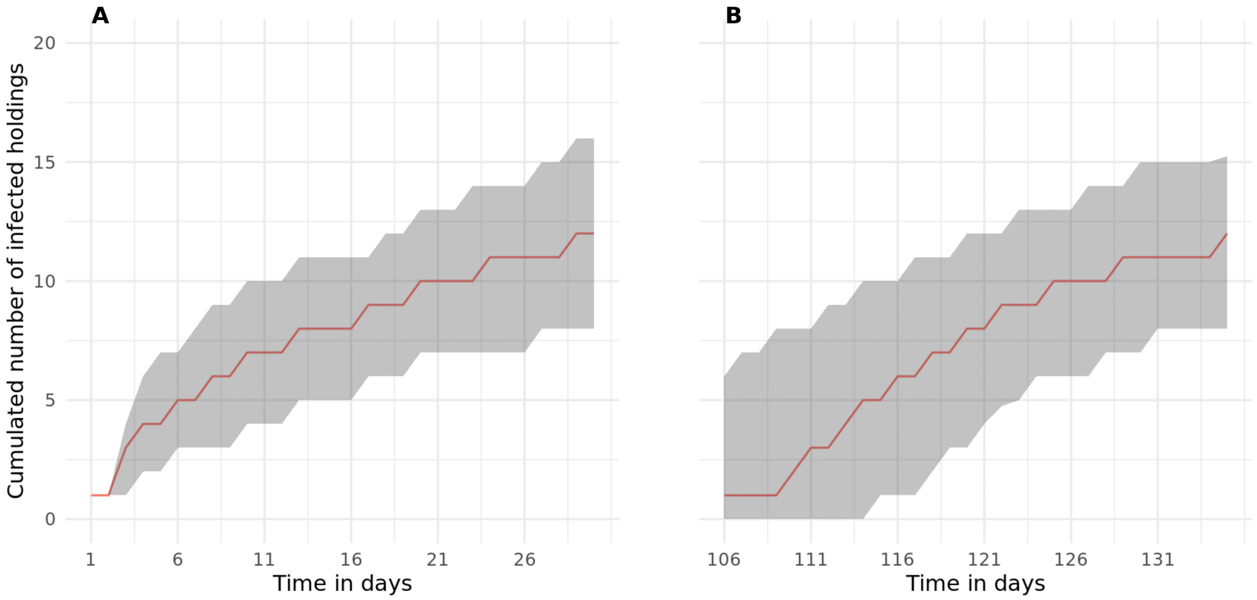

Figure 2: **Temporal dynamics of infected holdings in a 30-day simulation after hypothetical introduction of an ASF-like disease in Austria on (A) 1st of January and (B) April 2021.** One holding, located in the municipality of M1514(the highest-import municipality), was randomly selected as the origin of the outbreak. The red line represents the cumulative median count of infected holdings at each time point. The shaded gray area indicates the 95% confidence intervals (CIs), showing variability across 1,000 simulations. A holding was considered infected if it contained at least one pig in either the exposed (E) or infectious (I) compartments. Once a holding becomes infected, it remains infected for the duration of the simulation. The wider CIs at later time points highlight increased uncertainty in the model's predictions as the epidemic progresses.

Infection jumps via long-distance trade

Simulations initiated in April revealed an average of 3.2 $\pm$ 3.7 (SD) infection jumps per run via long-distance trade. The median number of jumps was two, demonstrating marked variability with a minimum and maximum counts of 0 and 26, respectively. The median time to the initial long-distance infection jump was 242 days $\pm$ 75.

Additionally, we identified three specific time frames characterized by the highest frequency of long-distance infection jumps (main text Figure 7). The first peak occurred between $t = 239 - 246$ days, at a probability of 42.1% of all long-distance infection jumps. Two subsequent peaks were observed at $t = 344 - 351$ days (25.9%) and $t = 358 - 365$ days (51.4%). Notably, the final peak coincides with the end of the simulation period (day 365).

To investigate the drivers of these multimodal patterns, we performed Poisson regression analysis relating weekly infection jumps to weekly overall trade. A statistically significant negative relationship was observed (coefficient = -0.0001, p = 0.008). However, the model exhibits substantial overdispersion (Deviance = 7615.7, Pearson χ^2 = 9.50e+03), indicating that the overall trade pattern alone cannot fully explain the peak formation.

Generalized Linear Model Regression Results

Dep. Variable:	January_Infections	No. Observations:	52
Model:	GLM	Df Residuals:	50
Model Family:	Poisson	Df Model:	1
Link Function:	Log	Scale:	1.0000
Method:	IRLS	Log-Likelihood:	-3940.5
Date:	Fri, 14 Nov 2025	Deviance:	7615.7
Time:	15:34:00	Pearson chi2:	9.50e+03
No. Iterations:	5	Pseudo R-squ. (CS):	0.1257
Covariance Type:	nonrobust		

	coef	std err	z	P>|z|	[0.025	0.975]
const	5.0708	0.183	27.764	0.000	4.713	5.429
Total_trade	-0.0001	3.87e-05	-2.650	0.008	-0.000	-2.67e-05

Figure 3: **Poisson regression analysis of long-distance infection jumps versus overall trade events**. Results from a generalized linear model (GLM) with Poisson distribution examining the relationship between weekly counts of two variables (N=52 weeks).

References

1. Puspitarani, G.A., Fuchs, R., Fuchs, K., Ladinig, A., and Desvars-Larrive, A. (2023). Network analysis of pig movement data as an epidemiological tool: an austrian case study. Scientific Reports *13*, 9623. doi: 10.1038/s41598-023-36596-1.
