# Supplementary material for: Risk of exotic disease introduction and propagation in the Austrian swine trade network
Source: iScience. 2026 Feb 2;29(3):114868. doi: 10.1016/j.isci.2026.114868 (PMC12925288; doi:10.1016/j.isci.2026.114868)
Supplement: Document S1. Figures S1–S5 [file mmc1.pdf]

iScience, Volume *29*

Supplemental information

Risk of exotic disease introduction and propagation in the Austrian swine trade network

Gavrila Amadea Puspitarani, Hannah Schuster, Ewan Colman, and Amélie Desvars-Larrive

Supplemental Data

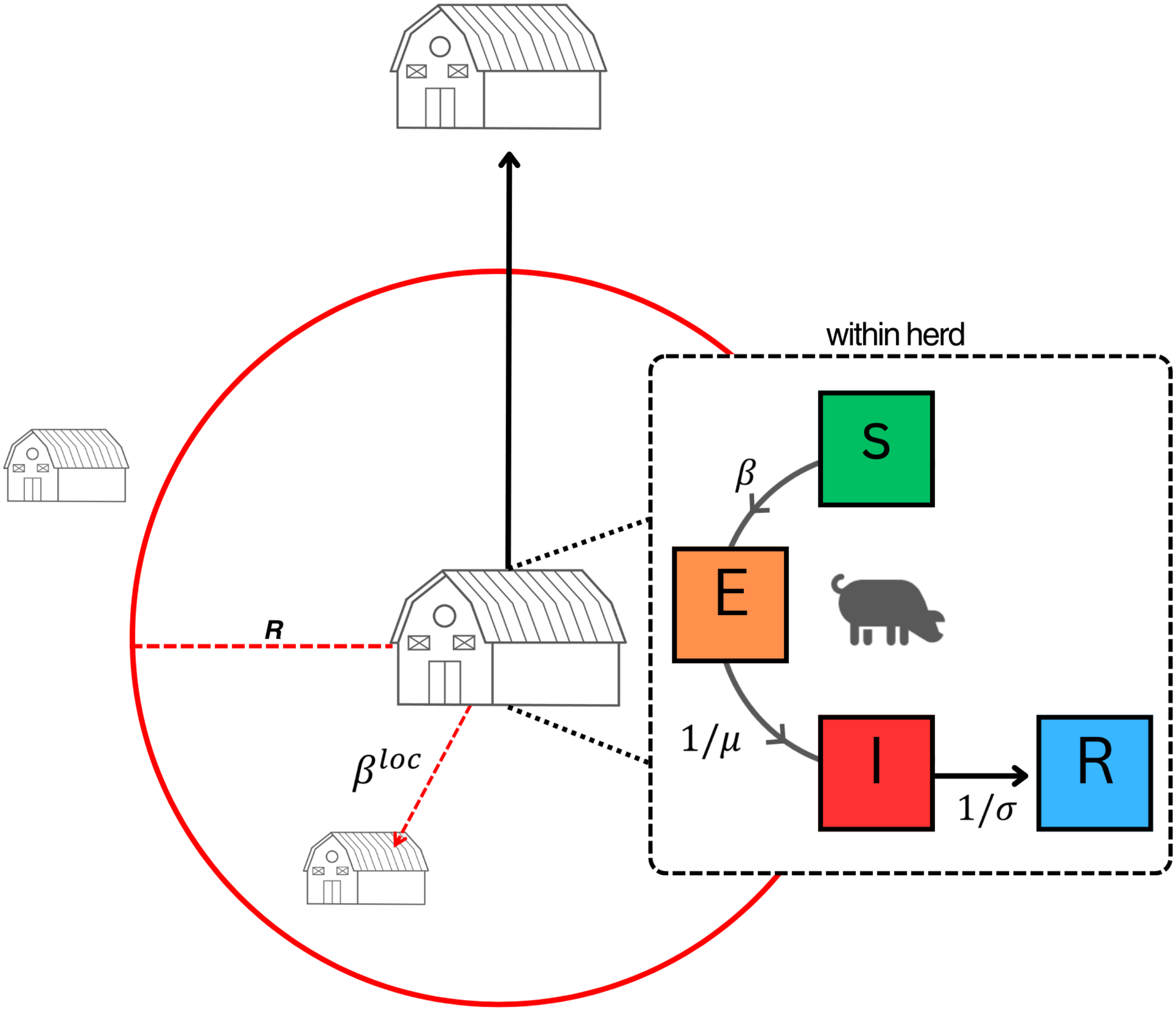

Figure 1: **Schematic representation of the epidemiological model.** The black dashed box represents a within herd stochastic Susceptible-Exposed-Infected-Removed (SEIR) transmission model in a homogeneous population. Black solid straight lines indicate direct trade connections recorded from movement data, while dashed red arrows represent a localized transmission process where an infected holding exerts a force of infection (β^{loc}) on susceptible holdings located within a 5-km radius (R). The red circle highlights the localized contagion area, illustrating the spatial constraints of the localized transmission process.

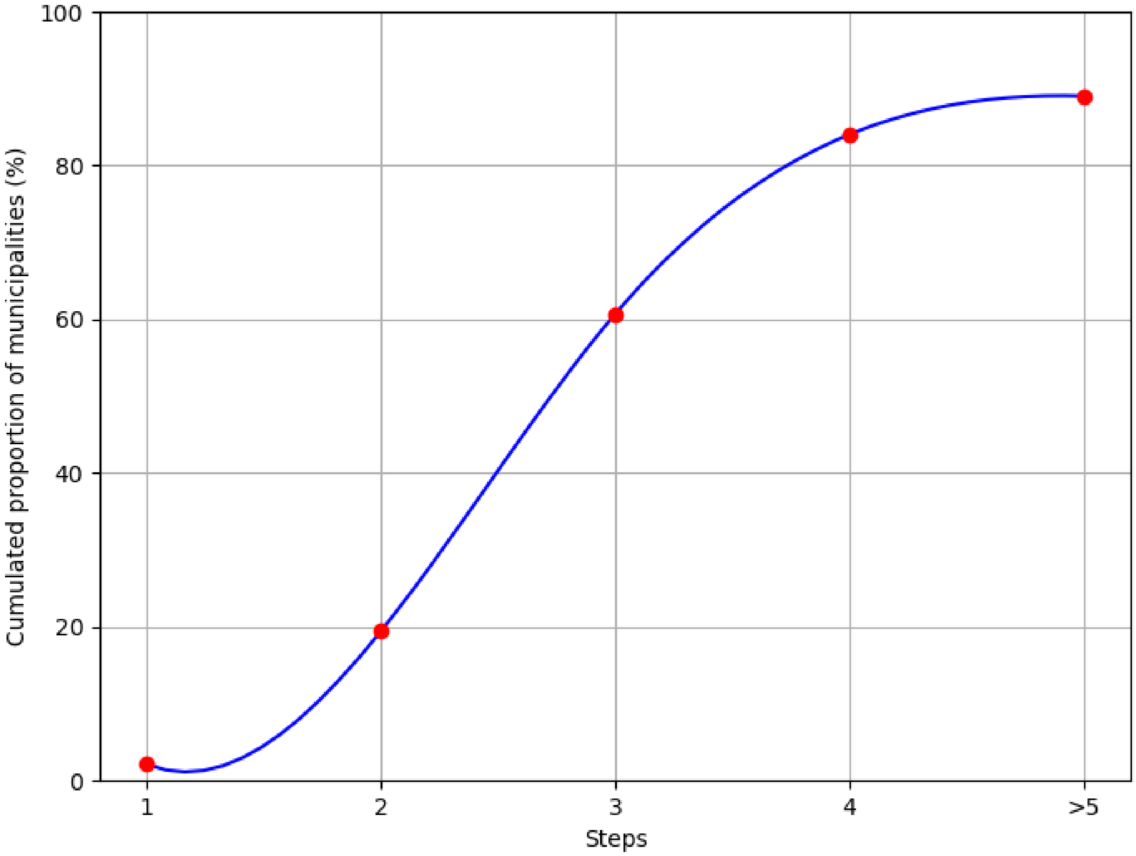

Figure 2: **Cumulative proportion of connected municipalities at each step of the potential propagation network G' from municipality M1514 (referred to as the origin).** Here, G' is a static network. A "propagation step" corresponds to a single trade movement between two connected municipalities within G', where step k corresponds to a trade between a municipality of order $k-1$ and one of order k. The x-axis represents the number of steps, with > 5 indicating connections beyond five steps; the y-axis shows the cumulative proportion of municipalities as a percentage of the total 2,115 municipalities in Austria.

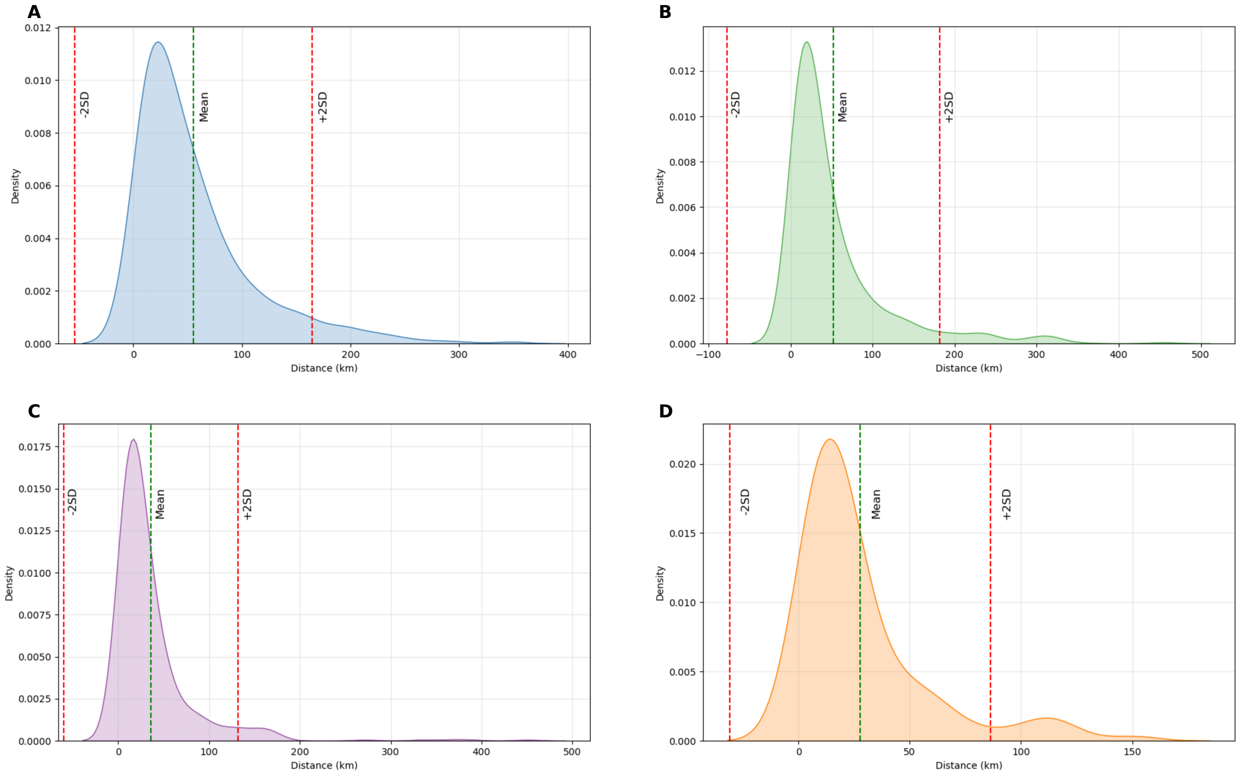

Figure 3: **Euclidean distance distribution between municipalities in the potential propagation network G' from municipality M1514 (referred to as the origin) at $k = 2$ to $k = 5$.** G' is a static network. The green dashed lines show the mean distance for each step. **A** Euclidean distance distribution for step 2 ($k = 2$), mean $=$ 55.5 $\pm$54.6 km. **B** Euclidean distance distribution for step 3 ($k = 3$), mean $=$ 52.2 $\pm$64.7 km. **C** Euclidean distance distribution for step 4 ($k = 4$), mean $=$ 35.9 $\pm$47.9 km). **D** Euclidean distance distribution for step 5 ($k = 5$) and beyond), mean $=$ 27.7 $\pm$29.3.

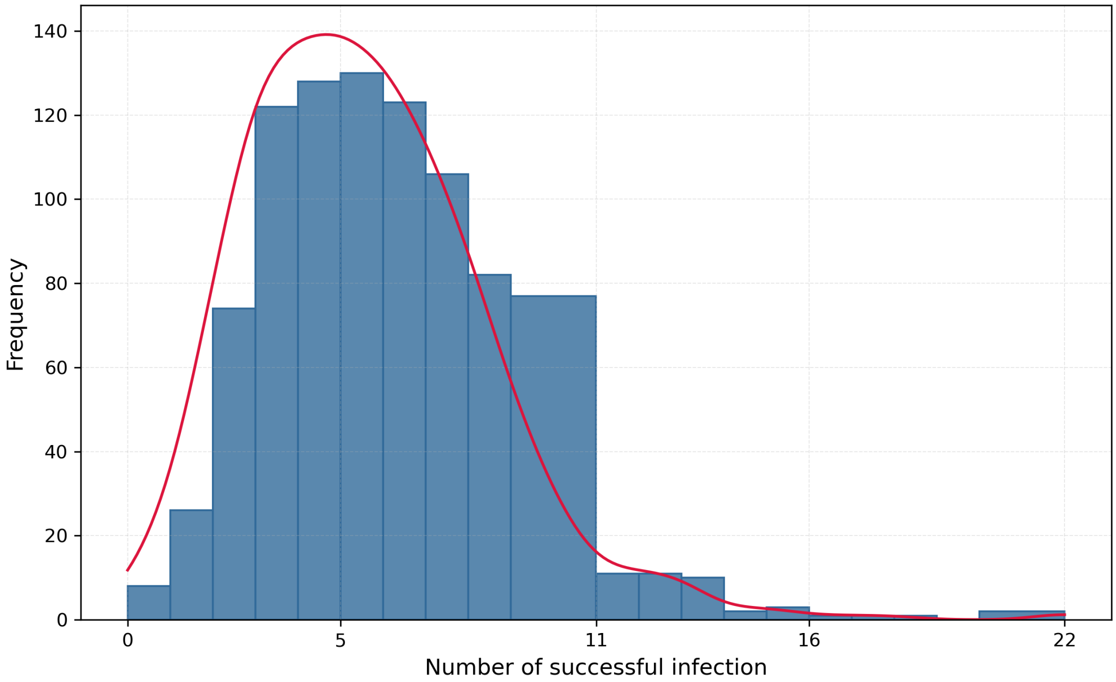

Figure 4: **Frequency of successful infections via long-distance trades after hypothetical introduction of an ASF-like disease in Austria on 1, January 2021.** One holding, located in the municipality of municipality M1514 (the highest-import municipality), was randomly selected as the origin of the outbreak. The histogram shows the distribution of successful long-distance infection jumps (i.e., $\geq$ 161.4 km), with an overlaid kernel density estimation (KDE) curve (red line). The mean number of occurrences of successful long-distance infection jumps per simulation was 5.6 $\pm$2.8 (SD).

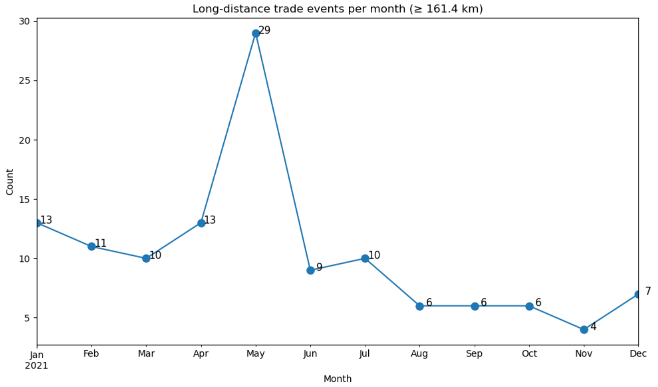

Figure 5: **Monthly distribution of long-distance trade events (i.e., $\geq$ 161.4 km) in Austria, 2021.** Values represent event counts per month, ranging from 4 (November)to 29 (December), with January and April each having 13 long-distance events. May had the highest frequency of long-distance trade events.
